# Evolution of warming tolerance alters physiology and life history traits in zebrafish

**DOI:** 10.1038/s41558-025-02332-y

**Published:** 2025-05-14

**Authors:** Anna H. Andreassen, Jeff C. Clements, Rachael Morgan, Davide Spatafora, Moa Metz, Eirik R. Åsheim, Christophe Pélabon, Fredrik Jutfelt

**Affiliations:** 1 Department of Biology, Faculty of Natural Sciences, Norwegian University of Science and Technology, Trondheim, Norway; 2 DTU Aqua: National Institute of Aquatic Resources, Technical University of Denmark, Kongens Lyngby, Denmark; 3 Fisheries and Oceans Canada, Gulf Fisheries Centre, Moncton, New Brunswick Canada; 4 Department of Biological Science, University of Bergen, Bergen, Norway; 5 Department of Integrative Marine Ecology, Sicily, Stazione Zoologica Anton Dohrn, Lungomare Cristoforo Colombo, Palermo, Italy; 6 National Biodiversity Future Center, Palermo, Italy; 7 Organismal and Evolutionary Biology Research Programme, Faculty of Biological and Environmental Sciences, University of Helsinki, Helsinki, Finland; 8 Centre of Biodiversity Dynamics, Department of Biology, Faculty of Natural Sciences, Norwegian University of Science and Technology, Trondheim, Norway; 9 Department of Biological and Environmental Sciences, Faculty of Science, University of Gothenburg, Gothenburg, Sweden

**Keywords:** Evolutionary ecology, Behavioural ecology, Ecophysiology

## Abstract

Evolution of warming tolerance may help species resist the impacts of climate change but can also lead to negative fitness outcomes. Identifying correlated responses to warming tolerance evolution could identify such negative consequences and help uncover the underlying mechanisms. By assessing the correlated responses of life history and physiological traits to seven generations of artificial selection to increase or decrease the acute upper thermal tolerance limit (CT_max_) in zebrafish (*Danio rerio*), we show that warming-adapted lines have improved cooling tolerance. Furthermore, the absence of difference between selected lines in aerobic metabolic scope, brain heat shock protein levels, fecundity, growth or swimming speed contradicts several hypotheses concerning the mechanisms controlling acute warming tolerance. These results suggest that selection due to acute heating events does not target variation in metabolic rates but can benefit tolerance to cold, making individuals more resilient to extreme temperature events.

## Abstract (web summary)

Using seven generations of selected zebrafish (*Danio rerio*), the authors consider the trade-offs and mechanisms behind evolution of warming tolerance. They show unexpected improvements in cooling tolerance in warming-adapted fish, and highlight mechanistic insights behind warming tolerance.

## Main

Fish and other ectotherms living in lakes, rivers and shallow coastal habitats can be vulnerable to rapid temperature change^1,2^. Species living at temperatures close to their thermal limits are especially vulnerable to further warming, such as warm-adapted ectotherms in tropical habitats^3^. This vulnerability is of particular concern in the light of climate change, as rapid extreme heating events are projected to more frequently exceed the physiological limits of many ectothermic species. Heat waves can lead to mass mortality events and affect the distribution of aquatic ectotherms^4–7^. These effects can be mitigated by the evolution of warming tolerance in response to increasing selection due to intensifying heat waves^8^.

Warming affects ectotherms by increasing the rates of chemical reactions and altering the shape and movements of molecules^9,10^. At the level of the organism, disrupted muscle or neural function leads to loss of balance or muscle coordination at the acute upper thermal limit, the highest temperature an animal can tolerate during rapid warming^11–15^. This warming tolerance may evolve, but the rate of the process and its potential for mitigating impacts depend on the underlying mechanisms^3^. However, the few empirical studies on the evolution of warming tolerance that exist indicate that this is a slow process with limited potential for evolutionary rescue from climate change^16–19^. Additionally, it remains uncertain whether and how such an evolution affects genetically correlated traits and ultimately the performance of affected populations^16^. While it is unknown which traits are altered during the evolution of warming tolerance, some hypotheses exist^13^.

**Fig. 1:**
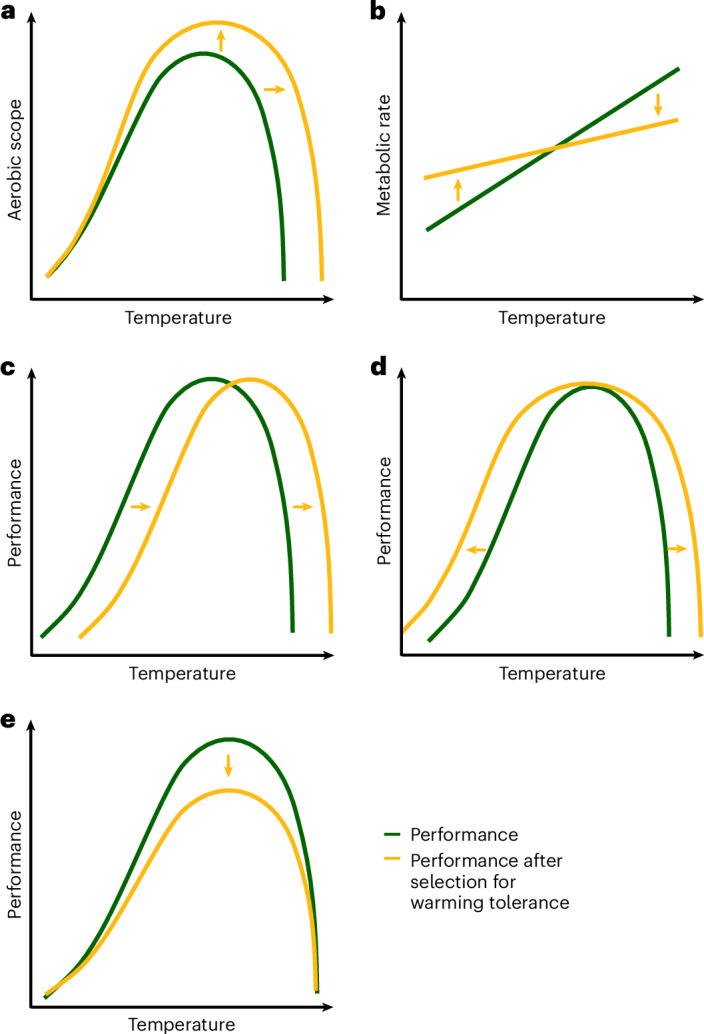
Different mechanisms may be responsible for the evolution of warming tolerance. **a**–**e**, These different mechanisms may be distinguished by their specific effects on various phenotypic traits (yellow lines). Changes in warming tolerance may result from increased oxygen supply capacity (**a**) or lower thermal sensitivity of metabolic traits (**b**). Changes in the thermal optimum of molecules may affect cell membrane fluidity and enzyme function leading to a shift in thermal optimum and warming tolerance (**c**). Selection for warming tolerance may lead to increased expression of heat shock proteins protecting against thermal extremes and may increase performance at both warming and cooling extremes (**d**). Trade-offs with warming tolerance may lower performance, such as growth or reproduction, when warming tolerance increases (**e**).

Increased chemical reaction rates and biological processes with warming will in turn increase the tissue oxygen demand. This may limit warming tolerance if oxygen supply does not increase at the same rate as demand because tissue hypoxia and ATP deficiency lead to muscle or neural failure^13,20^. Accordingly, the hypothesis of oxygen- and capacity-limited thermal tolerance proposes that limitations in the cardiorespiratory performance set the warming tolerance limits of ectotherms^21^. This hypothesis thus posits that animals with higher aerobic scope (that is, metabolic capacity above basal metabolic requirements) would be favoured during extreme heat challenges (Fig. 1a) because their oxygen supply is better able to meet increased metabolic demands^21^.

Alternatively, individuals with less thermally sensitive metabolic rates (for example, higher or faster acclimation capacity) could also be favoured by selection under extreme temperatures^22,23^. In this case, more warming-tolerant animals should be characterized by a lower increase in oxygen demand with increasing temperature (Fig. 1b).

Warming tolerance could also be limited by increased molecular movements and disruption of molecular bonds at high temperatures^24,25^. Both cell membrane and enzyme function rely on thermally sensitive molecular interactions. Increasing temperatures can make membranes more fluid and increase leakage^13^. If the evolution of warming tolerance promotes more rigid membranes that maintain function at high temperatures (homeoviscous adaptation), this can come at the cost of poorer function at cold temperatures where membranes are too rigid to function properly. Under such a scenario, we expect an upward shift in the thermal tolerance scope, the temperature range that the animal can acutely tolerate (Fig. 1c). A similar shift may occur if the evolution of warming tolerance is driven by an upward shift in optimal temperature for enzyme function. In this case, enzymes may perform better at higher temperatures but exhibit reduced efficiency and lower tolerance at cold temperatures. Occurrence of a shift in the thermal tolerance scope can be tested by measuring the cooling tolerance in populations that have evolved higher warming tolerance.

Alternatively, evolution of increased warming tolerance may improve tolerance to other stressors and expand the thermal tolerance scope (Fig. 1d) if, for example, it increases expression of heat shock proteins (HSP) that are critical for maintaining enzyme function during stress imposed by both extreme warming and cooling^26,27^. Testing this prediction can also be achieved by assessing the cooling tolerance of populations with evolved increased warming tolerance or by comparing their expression of HSP with lines selected to different warming conditions.

Finally, evolution of higher warming tolerance may decrease fitness under favourable or stable conditions if antagonistic pleiotropy affects warming tolerance and various performances (Fig. 1e). To assess these trade-offs, life history traits such as reproductive output or growth can be compared among populations adapted to different warming regimes.

We tested these predictions on the correlated responses to evolution of warming tolerance by examining the effects of selection for acute warming tolerance in zebrafish (*Danio rerio*) on a series of physiological and life history traits. With its short generation time, this tropical species inhabiting shallow waters that reach temperatures only a few degrees below its warming tolerance is particularly well suited to address questions about the evolution of warming tolerence^19,28,29^.

In this study, we used fish previously selected to increase or decrease the critical thermal maximum (CT_max_), the temperature at which individuals lose equilibrium during a rapid thermal ramping (0.3 °C min^−1^)^19^. Using the seventh and final generation of the artificial selection experiment, where CT_max_ had evolved towards higher and lower values, we assessed the correlated response of a range of traits including reproductive success, growth, thermal sensitivity of metabolic rates, aerobic metabolic scope, thermal tolerance scope and HSP expression. Physiological traits were selected to identify mechanisms suggested to limit acute warming tolerance, while life history traits were expected to reveal potential fitness costs to the evolution of warming tolerance.

## Warming-adapted lines have improved cooling tolerance

**Fig. 2:**
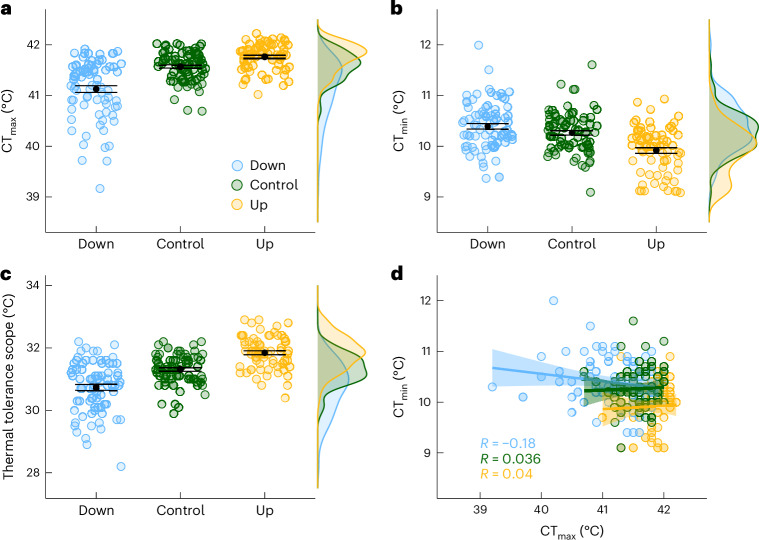
Thermal tolerance of fish artificially selected for warming tolerance. **a**, Acute upper thermal tolerance limit (CT_max_; *n* = individuals: Down = 78, Control = 78, Up = 70). **b**, Acute lower thermal tolerance limit (CT_min_; *n* = individuals: Down = 78, Control = 77, Up = 70). **c**, Thermal tolerance scope (CT_max_ − CT_min_; *n* = individuals: Down = 78, Control = 77, Up = 70). **d**, Correlation between CT_max_ and CT_min_. Data from the two measurement periods are pooled. Data are presented with mean (black points) ± s.e. (error bars).

We examined the correlated responses in a range of traits of zebrafish lines that had been artificially selected over seven generations for increased (Up-selected) or decreased (Down-selected) acute upper thermal tolerance (CT_max_) by comparing these to the control lines (Control). After seven generations of selection, CT_max_ had changed more in the Down-selected treatment than in the Up-selected treatment. Despite an interaction effect between selection treatment and the measurement period (see Supplementary Table 3 for model selection and methods for explanation of the period), fish from the Down-selected treatment consistently displayed lower CT_max_ than fish from the Control treatment (period 1: −0.67 °C, 95% confidence interval (CI): −0.91 to −0.43; period 2: −0.31 °C, 95% CI: −0.53 to −0.09). Although fish from the Up-selected treatment consistently displayed higher CT_max_ than in the Control treatment (period 1: 0.22 °C, 95% CI: −0.02 to 0.46; period 2: 0.17 °C, 95% CI: −0.03 to 0.37), the absolute difference with the Control treatment was weaker than for the Down-selected treatment (Figs. 2a and 5). The CT_max_ in the Down-selected treatment was also more variable than in the two other selection treatments (mean; s.d. Down_1_: 41.1 °C; 0.62 °C; and Down_2_: 41.2 °C; 0.55 °C; Control_1_: 41.7 °C; 0.35 °C; and Control_2_: 41.5 °C; 0.19 °C; Up_1_: 41.8 °C; 0.27 °C; and Up_2_: 41.7 °C; 0.27 °C; Fig. 2a). These results are similar to those reported in ref. ^19^ at the sixth generation.

**Extended Data Fig. 1:**
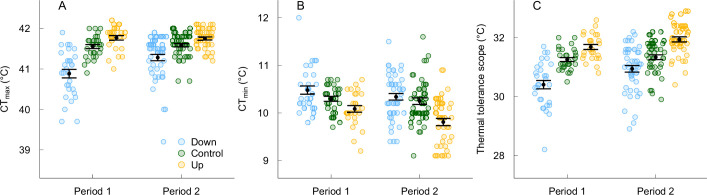
Effect of sampling period on thermal tolerance for the three selection treatments. Due to the Covid-19 outbreak, the fish from each tank were phenotyped over two experimental periods, and the data are separated by phenotyping period. **a** Acute upper thermal tolerance limit (CT_max_; *n* = individuals in Period 1: Down = 30, Control = 30, Up = 27; and in Period 2: Down = 48, Control = 48, Up = 43), **b** Acute lower thermal tolerance limit (CT_min_; *n* = individuals in Period 1: Down = 30, Control = 30, Up = 27; and in Period 2: Down = 48, Control = 47, Up = 43), **c** Thermal tolerance scope (CT_max_ – CT_min_; *n* = individuals in Period 1: Down = 30, Control = 30, Up = 27; and in Period 2: Down = 48, Control = 47, Up = 43). The fish in period 1 were sampled at 74–76 dpf and the fish in period 2 at 98–100 dpf (Supplementary Fig. 4).

During the first measurement period, the Up-selected treatment had an acute cooling tolerance (CT_min_) that was lower than the Control treatment (−0.35 °C, 95% CI: −0.62 to −0.08). However, we found no statistical difference between the Down-selected and the Control treatments (0.13 °C, 95% CI: −0.14 to 0.40; Figs. 2b and 5 and Supplementary Table 4). During the second measurement period, CT_min_ was on average −0.16 °C (95% CI: −0.28 to 0.05) lower than in the first period in all treatments, but the differences among selection treatments were similar to those observed in the first period (Extended Data Fig. 1 and Supplementary Table 3).

Combining the evolutionary response of CT_min_ and CT_max_ showed that, compared to the Control treatment, Up-selected fish expanded the temperature range they could acutely tolerate, while the Down-selected treatment reduced this range. For the Up-selected treatment, the range was 0.43 °C (95% CI: 0.04 to 0.82) and 0.60 °C (95% CI: 0.27 to 0.93) wider than the Control treatment in the first and second period, respectively, while the Down-selected treatment showed a narrower range decreasing by −0.84 °C (95% CI: −1.21 to −0.47) and −0.40 °C (95% CI: −0.73 to −0.07) in the first and second time periods, respectively (Figs. 2c and 5 and Supplementary Table 4). We found no correlation between individuals’ CT_min_ and CT_max_ in any of the selection treatments (Fig. 2d).

## Increased warming tolerance is not related to oxygen uptake

**Fig. 3:**
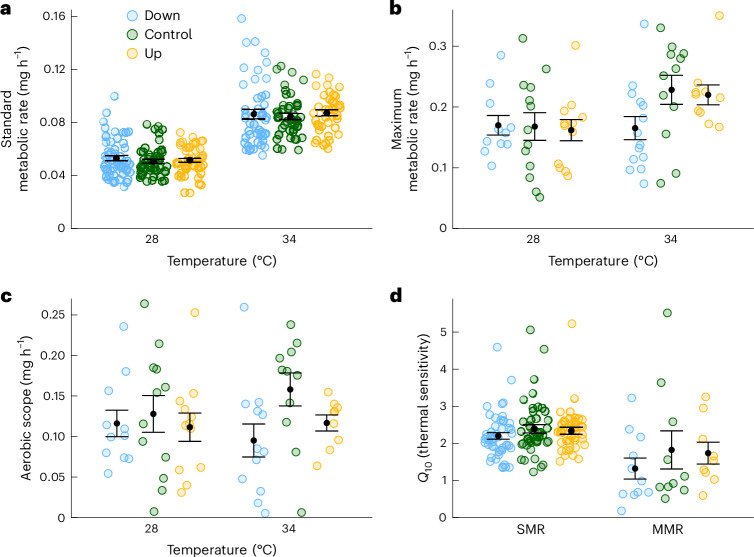
Effects of selection treatment and temperature on metabolic rates. **a**, SMR of individuals (*n* = individuals at 28 °C: Down = 58, Control = 52, Up = 50; and at 34 °C: Down = 49, Control = 44, Up = 41). **b**, MMR estimated as average MMR per individual measured in groups of three to four fish (*n* = groups at 28 °C: Down = 11, Control = 13, Up = 12; and at 34 °C: Down = 14, Control = 12, Up = 10). Both SMR and MMR are adjusted to a mean mass of 105 mg. **c**, Aerobic metabolic scope (MMR − SMR; *n* = groups at 28 °C: Down = 11, Control = 13, Up = 12; and at 34 °C: Down = 13, Control = 12, Up = 9). **d**, Metabolic response to temperature (*Q*_10_) of SMR (*n* = individuals: Down = 49, Control = 44, Up = 41) and MMR (*n* = groups: Down = 11, Control = 10, Up = 9). Data from the two measurement periods are pooled. Data are presented with mean (black points) ± s.e. (error bars).

Standard metabolic rate (SMR) at normal holding temperature (28 °C) or after 3 hours of exposure to an elevated temperature (34 °C) did not differ between selection treatments (Figs. 3a and 5 and Supplementary Tables 5 and 6). In contrast, the maximum metabolic rate (MMR) differed between treatments when measured at 34 °C, but not at 28 °C (Figs. 3b and 5 and Supplementary Table 5). This effect was mainly caused by a decrease in MMR by −0.06 mg h^−1^ (95% CI: −0.12 to −0.01) in the Down-selected treatment compared to the Control treatment, while MMR did not differ between the Up-selected and the Control treatment (−0.01 mg h^−1^, 95% CI: −0.07 to 0.05; Supplementary Tables 7 and 8). Differences in MMR resulted in a narrower aerobic scope (−0.06 mg h^−1^, 95% CI: −0.12 to −0.01) in the Down-selected treatment compared to the Control treatment at 34 °C, while the difference between the Up-selected and Control treatment was small and statistically not significant (−0.03 mg h^−1^, 95% CI: −0.09 to 0.03; Figs. 3c and 5 and Supplementary Tables 7 and 8). We did not detect an effect of selection treatment on individuals’ factorial change in rate over 10 °C (*Q*_10_) of SMR or MMR due to the similar response of both SMR and MMR to elevated temperature in all selection treatments (Figs. 3d and 5 and Supplementary Tables 5 and 7).

## No trade-offs detected between warming tolerance and life history traits

**Fig. 4:**
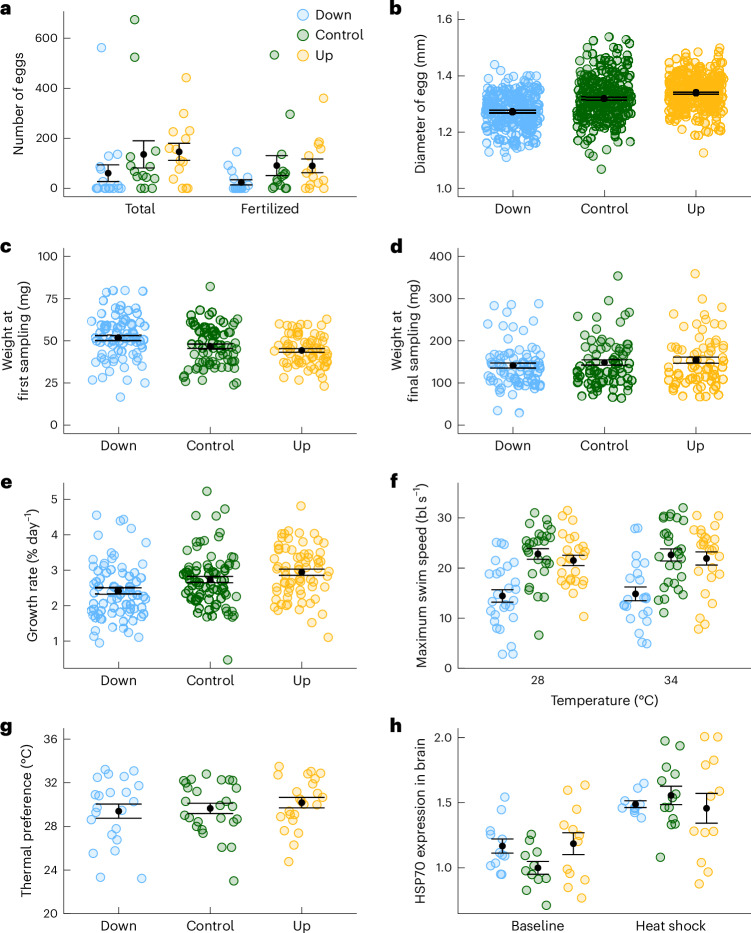
Effects of warming tolerance selection on life history and physiological traits. **a**, Number of eggs from spawning boxes (*n* = boxes: Down = 17, Control = 14, Up = 14) during the initial round of reproduction. **b**, Egg diameter of a subset of ten eggs from each box (*n* = eggs: Down = 223, Control = 263, Up = 287). **c**, Initial weight (*n* = individuals at 44–47 dpf: Down = 78, Control = 78, Up = 70). **d**, Final weight (*n* *=* individuals at 74–100 dpf: Down = 78, Control = 78, Up = 70). **e**, Specific growth rate (*n* = individuals: Down = 77, Control = 78, Up = 70). **f**, Maximum swim speed (*n* = individuals at 28 °C: Down = 27, Control = 28, Up = 25; and at 34 °C: Down = 22, Control = 27, Up = 25). **g**, Thermal preference (*n* = individuals: Down = 22, Control = 26, Up = 23). Data from the two measurement periods are pooled (**d**–**g**). **h**, Brain HSP70 level relative to the Control treatment at baseline (*n* = individuals at baseline: Down = 12, Control = 11, Up = 12; and after heat shock: Down = 10, Control = 13, Up = 12). Data are presented with mean (black points) ± s.e. (error bars).

**Fig. 5:**
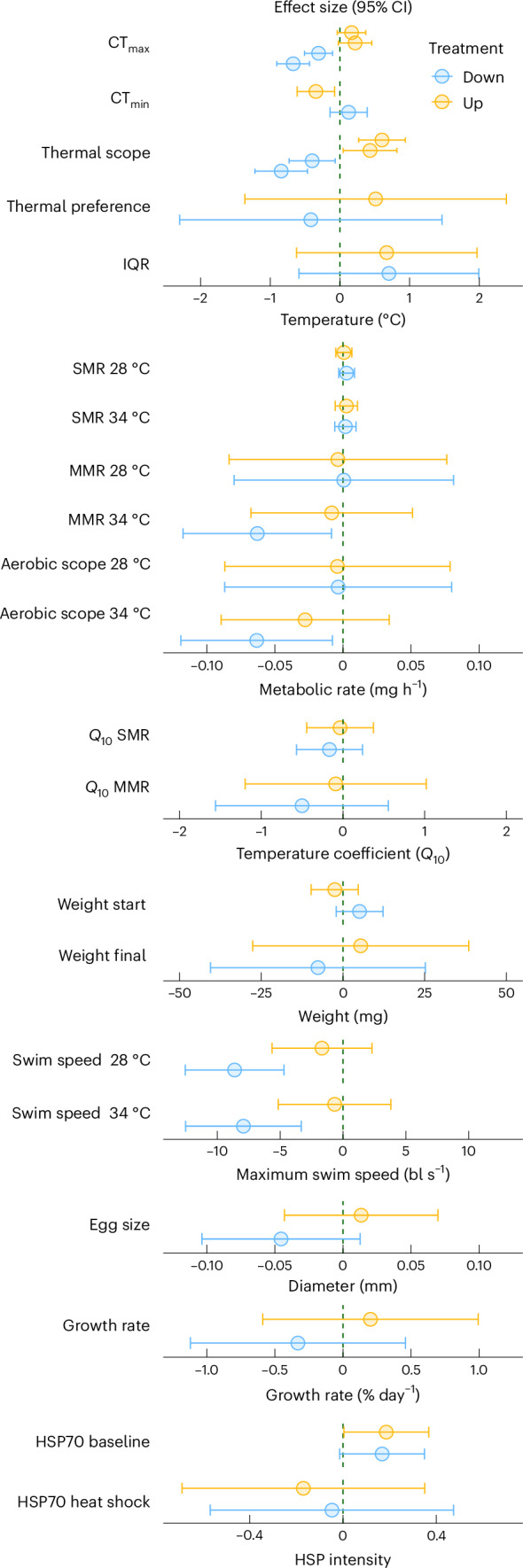
Effect of selection for acute upper thermal tolerance in Up-selected (yellow) or Down-selected (blue) fish. Effect sizes are presented as difference from the Control treatment (dashed line in the centre) with their 95% CI (error bars) obtained from models including selection treatment as fixed factor. IQR, interquartile range of occupied temperature in a thermal gradient.

**Extended Data Fig. 2:**
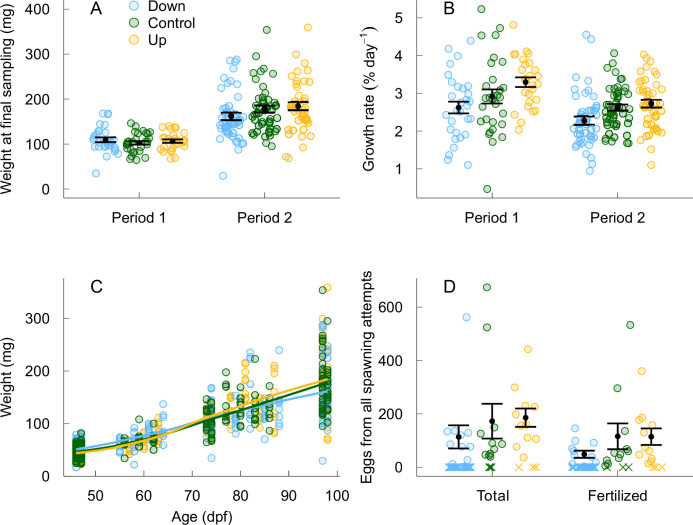
Effects of selection treatments on reproduction and growth. **a** Fish weight at final sampling (*n* = individuals in Period 1: Down = 30, Control = 30, Up = 27; and in Period 2: Down = 48, Control = 48, Up = 43), and **b** growth rate (*n* = individuals in Period 1: Down = 30, Control = 30, Up = 27; and in Period 2: Down = 47, Control = 48, Up = 43), separated by phenotyping period (1 and 2). The fish in period 1 were sampled at 74–76 dpf and the fish in period 2 at 98–100 dpf (Supplementary Fig. 4). **c** All weight measurements fitted with local polynomial regression (*loess*) (*n* = individuals: Down = 78, Control = 78, Up = 70). **d** Number of eggs from all spawning attempts including additional rounds of reproduction of the Down-selected lines. The means and standard error are presented for the successful boxes (*n* = spawning boxes with eggs: Down = 12, Control = 12, Up = 11), excluding boxes with zero eggs represented as crosses (*n* = spawning boxes without eggs: Down = 31, Control = 3, Up = 3).

During the first round of reproduction, only 41% of spawning attempts (7 of 17) were successful in the Down-selected treatment while 79% were successful in both the Control and Up-selected treatments (11 of 14 in each). Consequently, we set up additional reproduction boxes for the Down-selected lines (Figs. 4a and 5, Supplementary Table 1 and Extended Data Fig. 2). Egg volume was on average 10% smaller in the Down-selected treatment (difference in diameter: −0.05 mm, 95% CI: −0.10 to 0.01; Figs. 4b and 5 and Supplementary Tables 9 and 10) and 3% larger in the Up-selected compared with the Control treatment (difference in diameter: 0.01 mm, 95% CI: −0.04 to 0.07), although these differences had low statistical support (95% CI overlapped with zero). This result contrasts with the weight of the juveniles at 44–47 days post fertilization (dpf), where fish from the Down-selected treatment were on average 11% heavier than the Control fish (5.10 mg, CI 95%: −2.06 to 12.24), while the fish from the Up-selected treatment weighed 5% less than the Control (−2.54 mg, CI 95%: −9.75 to 4.67; Figs. 4c and 5 and Supplementary Tables 9 and 10; see Supplementary Fig. 1 for length differences). These differences, however, disappeared at 74–76 and 98–100 dpf when no differences in fish weight were observed between selection treatments (model selection favoured a model without treatment; Supplementary Table 9). This suggests compensatory growth in the Control and Up-selected treatments, although differences between treatments in specific growth rates were not statistically significant (Figs. 4d,e and 5, Supplementary Fig. 4 and Supplementary Tables 9 and 11; see Supplementary Fig. 2 for the relationship between the first and the final weight measurements).

## Warming tolerance is not related to warming performance at higher temperatures

Maximum swimming speed was 41% and 36% slower during the first and second measurement periods, respectively, in Down-selected fish compared to the Control fish at normal temperature (−8.62 body lengths per second (bl s^−1^), 95% CI: −12.56 to −4.68) and 35% slower at elevated temperatures (average for both periods: −7.92 bl s^−1^, 95% CI: −12.53 to −3.31; Figs. 4f and 5 and Supplementary Tables 12 and 13; see Supplementary Fig. 1 for group swimming speed). Swimming speeds at 28 °C and 34 °C were correlated among fish in the Control and Up-selected treatments, but not among Down-selected fish (Supplementary Fig. 2c). Finally, we did not observe any effect of the selection treatment on the thermal preference of individuals exposed to a thermal gradient (Figs. 4 and 5 and Supplementary Tables 12 and 14; see Supplementary Fig. 1 for the thermal range).

## No detected change in the heat shock response

We found no effect of selection on baseline HSP70 (70 kDa HSP) expression in brain tissue from fish sampled at holding temperatures of 28 °C or from fish exposed to a 1-hour heat shock (ramping at 0.3 °C min^−1^ to 38 °C and 10-min exposure to 38 °C; Figs. 4h and 5 and Supplementary Tables 12, 15 and 16).

## Discussion

If evolution of warming tolerance results from the evolution of membranes or proteins with higher thermal optima, we would expect reduced performance of some functions at low temperatures (Table 1). Contrary to this prediction, the lines with increased warming tolerance also showed increased tolerance to cold water. Consequently, the Up-selected fish evolved larger thermal windows. This suggests that the evolution of heat-tolerant membranes and/or proteins is an unlikely explanation for acute warming tolerance evolution, despite this mechanism often being suggested to underlie adaptation to chronically warmer temperatures (for example, ref. ^30^). The improvement of both cold and warm tolerance is instead expected with the evolution of stronger heat shock responses because HSP may protect the function of enzymes during both warm and cold challenges^26,27^. The evolved mechanisms may include higher constitutive levels, increased production, or faster induction of HSP expression during the thermal challenges^31,32^ (Table 1).

**Table 1:** Predicted and observed response to selection for increased warming tolerance for mechanisms hypothesized to limit warming tolerance

Limiting mechanism	Predicted response	Observed response
Oxygen supply capacity	Increased aerobic scope (Fig. 1a)	No (Fig. 3c)
	Decreased *Q*_10_ (Fig. 1b)	No (Fig. 3d)
Thermal optimum of molecules	Decreased CT_min_ (Fig. 1c)	No (Fig. 2b)
Heat shock response	Increased CT_min_ (Fig. 1d)	Yes (Fig. 2b)
	Increased HSP levels	No (Fig. 4h)

However, none of our measures of HSP70 expression (baseline level in the brain or the response in the brain within the time course of a CT_max_ test) showed differences among treatments that could explain the differences in thermal windows observed between the selection lines. Heat shock responses, however, often involve many different proteins expressed in various tissues. Therefore, our measurements of HSP70 in the brain may have missed differences in the expression of other HSPs in specific brain regions or in other organs important for acute thermal tolerance.

A main contender for the mechanism limiting warming tolerance is oxygen delivery to the body^13,33^. In our experiment, evolution of such mechanisms would have meant evolution of several metabolic traits. Our results do not support these predictions (Table 1). MMR and aerobic scope were not higher in the Up-selected fish than in the Control fish, demonstrating that the increase in warming tolerance was not caused by higher capacity for oxygen transport. This contradicts the assumption that oxygen transport capacity limits warming tolerance^21^ and is instead consistent with findings showing that other physiological systems can be the first to fail during warming^13,34,35^. Similarly, the thermal sensitivity (*Q*_10_) of metabolic rates remained unaffected by the selection for increased warming tolerance. This implies that a reduced thermal sensitivity of metabolic rates was not the cause of the elevated warming tolerance in the Up-selected lines. Together, these results suggest that the mechanisms responsible for the evolution of warming tolerance are mostly independent of metabolic traits and therefore not caused by adjustments in metabolic rates or their thermal sensitivity.

We did not detect trade-offs between the evolution of increased warming tolerance and physiological performance (Table 1). We also found no difference in growth or fecundity between the Up-selected and Control fish (Table 1). Previous studies found that the warming tolerance limit was conserved in zebrafish maintained for 150 generations in a thermally constant laboratory environment^28,36^. Together, these results suggest that warming tolerance is either not involved in genetic trade-offs with physiological and life history traits or is genetically linked to fitness traits that are also important in a laboratory environment. Note, however, that the good conditions encountered in the lab with constant temperature and ad libitum food supply may obscure trade-offs between performance and high warming tolerance that would be more apparent in nature.

In contrast, the Down-selected fish showed a reduction in spawning success, egg size, maximum swimming speed at both normal and elevated temperatures, and a lower MMR at higher temperatures compared to the Control and Up-selected lines. These reduced performances and the large variation in warming tolerance compared to the other lines suggest that Down-selected fish evolved lower overall performance. This could be due to pleiotropy between these performance traits and the traits contributing to the reduction in warming tolerance since many deleterious genetic effects could decrease both warming tolerance and performance. The Up-selected lines did not display higher performance in those traits than the Control lines. This asymmetry between the Up- and Down-selected lines in the correlated response of performance traits to selection on warming tolerance suggests that the underlying mechanisms for decreased warming tolerance in the Down-selected lines may be distinct from the mechanisms that caused the improvements in warming tolerance in the Up-selected lines. Alternatively, the asymmetry could result from a lower potential for increasing performance in traits that are already close to their maximum values in the Up-selected lines compared to the Down-selected lines. This could be the case in the presence of a hard ceiling for thermal tolerance traits^19,37^ and in the presence of strong trade-offs with other life history traits^38^.

Contrary to our predictions, evolution of warming tolerance in fish facing climate change-amplified heat waves can lead to increased tolerance to acute cooling. Thus, evolved warming tolerance may not necessarily reduce tolerance to winter cold spells, as previously assumed. The evolved expansion in thermal tolerance scope may therefore allow tolerating larger seasonal fluctuations resulting from climate change-induced poleward migration in fishes.

Although metabolic rates were not affected by the evolution of increased acute warming tolerance, they might still be under selective pressure from chronic warming exposures^33^. Yet, despite the important consequences that connections between various thermal traits could have on the evolutionary response to climate change, it remains uncertain whether key traits to resist rapid heating events are also important during chronically increased temperatures^6,39,40^. In our experiment, evolution of increased warming tolerance was not accompanied by improved swimming performance at increased temperatures, changes in metabolic rates or thermal preference. These results suggest that warming-tolerant individuals are not performing better at moderately elevated temperatures and that tolerance to rapid warming is not linked to thermoregulation. Hence, various thermal traits are likely to respond differently to the selection generated by acute or chronic increases in temperature.

How well adaptation to heat waves can be mimicked through artificial selection for CT_max_ is currently unknown^19,41^. However, the mechanisms underlying acute warming tolerance are likely to be similar across various acute thermal challenges^13^ and individual zebrafish that perform well in rapid CT_max_ also tolerate slower warming better than conspecifics^39^, indicating that adaptation to one acute warming scenario would translate across thermal challenges, including exposure to climate change-amplified heat waves in nature.

The relative importance of adaptation to warming that is acute (extreme heat waves) or chronic (elevated mean temperatures) might also depend on the species and their habitats. While our results suggest that the costs of improved warming tolerance are limited, they do not necessarily imply that the evolution of warming tolerance in zebrafish is easy and can be sustained over a large range of temperatures. Morgan et al.^19^ have shown that the evolution of increased warming tolerance in zebrafish is slow, and that the response is small compared to the pace of the current warming. They further suggested that the benefit of warm acclimation may diminish with the evolution of higher warming tolerance^19,36^. These results therefore support the idea that warm-adapted ectotherms might be among the species most susceptible to climate change-induced extinction because environmental temperatures may rapidly exceed their physiological limits for which acclimation and adaptive changes are limited^3^.

## Conclusion

We assessed how selection over seven generations for acute warming tolerance affected a range of physiological and life history traits. While acute warming tolerance clearly evolved, several predicted consequences of this evolution were not observed, therefore questioning the generality of the mechanisms suggested to underly the evolution of CT_max_. One of the most unexpected results, namely that the evolved increase in warming tolerance concurred with the expansion of cold tolerance, may have important ecological consequences for the distribution and phenology of fish species exposed to extreme heat waves.

## Methods

In this study, we used descendants of fish selected for seven generations towards higher or lower acute upper thermal tolerance^19^. We summarize the protocol of the artificial selection experiment (details in ref. ^19^), before presenting the methods specific to the current study.

### Ethics statement

The experimental procedures were conducted in line with the Norwegian Animal Welfare Act and the Regulation on the Use of Animals in Research, and the experiment was approved by the Norwegian Animal Research Authority (permit no. 8578).

### Breeding and fish husbandry

Zebrafish (*D. rerio*) from wild-caught populations from West Bengal, India, were brought into the lab at the Norwegian University of Science and Technology in 2016. These were reproduced in the lab to obtain the parental generation for the selection lines (F_0_). To start the breeding, groups of three females and three males were placed in spawning tanks (2.5 l) and left overnight at 26 °C. The spawning tanks contained a mesh on the bottom allowing the eggs to fall through and a plastic plant as shelter. Between 118 and 366 wild-caught fish contributed to this parental generation (F_0_, *n* = 1,200). After spawning, adults were removed, and at 2 dpf the eggs were transferred to new tanks with a 2-cm water column (0.5 l). Between 7 and 10 dpf, larvae were transferred to their final holding tanks (63 l) containing water filters, plastic plants and air supply, and connected to a flow-through system. Fish density was kept under five larvae per litre (300 in each tank). Fish were fed ad libitum three times per day, alternating between live Artemia sp., *zebrafeed* and GEMMA Micro (Skretting). Three weeks post fertilization, the holding temperature was increased to 28 ± 0.2 °C and the diet gradually transitioned to dry TetraPro flakes (Tetra) twice per day and Artemia sp. once per day.

### Artificial selection experiment

Selection for acute upper thermal tolerance limits started in September 2017 on juveniles from the F_0_ approximately six weeks post fertilization. The acute upper thermal tolerance limit was quantified by the critical thermal maximum (CT_max_) method^11^. This metric is repeatable and correlates with warming tolerance measured during slow ramping rates^15,39^. Although measurements of warming tolerance depend on heating rate, acclimation temperature and duration of exposure, warming tolerance is correlated to both distribution and extreme daily temperatures for aquatic ectotherms across methodological approaches^6^.

The CT_max_ was measured using the protocol presented below in the Acute thermal tolerance section. Control lines were started with 300 randomly selected fish before their CT_max_ was measured. These were divided into two replicate lines (*n* = 150 in each) with the same CT_max_ distributions. The remaining 900 fish were tested for CT_max_ and two replicate Down-selected lines were formed from fish with the 33% lowest CT_max_ values (*n* = 150 each) and two replicated Up-selected lines were formed from fish with the 33% highest CT_max_ values (*n* = 150 in each). Each of the six replicated lines was divided between two 63-l holding tanks (75 individuals in each) that were randomly placed in the animal holding room.

At maturity, approximately three months after fertilization, fish from this parental generation (F_0_) were bred using the same protocol as described above to produce 450 offspring per replicate line (F_1_ generation). In each line, all 450 fish were tested for CT_max_ and 150 fish were selected following the selection protocol of the Control, Down- and Up-selected lines. The study by Morgan et al.^19^ presents the results after six episodes of selection. We continued the selection for one more generation, the F_7_ generation, on which we performed the measurements for the current study, in January 2020.

### Egg size and number

Fish from the F_6_ generation were bred between 9 and 13 January 2020. All eggs were counted and identified as fertilized or unfertilized (Supplementary Table 1). Seven spawning boxes with three males and three females were set up for each replicated line (14 in each of the Control and Up-selected treatments, 17 in the Down-selected treatment where three additional boxes were set up for one of the replicated lines). Additional rounds of reproduction were set up if too few fertilized eggs were produced but, for consistency, only the initial breeding round was used for comparing egg number and the proportion of fertilized eggs between treatments (see Supplementary Table 1 for results from all spawning boxes). A subset of ten fertilized eggs from each successful breeding box was photographed at 3 dpf using a stereomicroscope, and the horizontal diameter of each egg was measured using ImageJ (National Institutes of Health, United States).

### Tagging

Six weeks after fertilization, on 25 and 26 February, 40 juvenile F_7_ fish from each of the six selected lines (*n* = 240) were tagged, weighed and their length measured. The fish were anaesthetized with a buffered solution of MS-222 (tricaine mesylate, 110 mg l^−1^) before they were tagged with visible implant elastomers (Northwest Marine Technologies, United States) in the dorsal muscle on both sides of the dorsal fin^42^. Immediately after tagging, they were weighed (±0.001 g) on a digital scale and the standard length (±0.01 mm) was measured using a digital calliper. Twenty fish from the same line with unique coloured tag combinations were housed together in 12 holding tanks for the remaining part of the experiment (Supplementary Fig. 3).

### Further phenotyping at F_7_

Sixteen out of the 20 fish from each tank were tested for various traits, leaving four extra fish per tank in case of mortality. Fish from the same tank were tested in groups of eight fish over a 4-day phenotyping period (Supplementary Fig. 3 and Supplementary Table 2). Due to the Covid-19 outbreak, the fish from each tank went through phenotyping over two experimental periods (29 February to 15 March and 24 March to 8 April). The groups were ordered to spread out selection treatments, replication lines and replication tanks, and ensured that eight fish from each tank were phenotyped before the lockdown; the remaining eight fish were tested 24 days later (Supplementary Figs. 3 and 4 and Supplementary Table 2).

Groups of eight fish were isolated and feeding stopped the day before phenotyping started. On the first phenotyping day, the thermal preference was measured for four of the eight fish. On the second day, all eight fish were put in respirometers to measure resting oxygen consumption overnight at a holding temperature (28 °C). The following morning the oxygen consumption at an elevated temperature (34 °C) was measured, followed by measurements of the individuals’ weight and length. On the fourth day of phenotyping, the maximum oxygen consumption and maximum swim speed were recorded at 28 °C and 34 °C before the fish were returned to their holding tanks and the feeding and normal holding routines resumed. One group was started every day, so it took 15 days to complete the phenotyping for the 12 groups of eight fish during both periods. Five days after the last group had gone through the phenotyping, we started a final sampling of acute thermal tolerance limits, and we recorded weight and length (Supplementary Fig. 4 and Supplementary Table 2).

### Thermal preference

We tested the thermal preference of the selected fish in an annular arena with a thermal gradient ranging from 22 °C to 35 °C (ref. ^43^). The day before the test, four fish were moved to individual tanks with the same shape as the preference arena for habituation. The habituation tanks were aerated and kept at holding temperature (28 ± 0.02 °C). On the day of testing, fish were tested individually for thermal preference by gently transferring them into the thermal gradient at one of the positions with an intermediate temperature close to the holding temperature. A video recording started right after the fish was placed in the arena using a USB camera (C1 Kurokesu) logging to OBS Studio (Open Broadcaster Software). The thermal gradient was monitored and recorded every two seconds using 24 type K thermocouples (10 m, Pico Technology) placed along the walls of the arena and connected to thermocouple data loggers (TC-08, Pico Technology). Each fish was tracked for 2 hours from the video recordings using EthoVision (XT 13.0, Noldus IT) and its position in the arena was matched with the corresponding recorded temperature. Once the trial ended, the fish was moved to a tank with the rest of its group. To allow time for the fish to habituate to the setup, thermal preference was estimated as the median temperature occupied by the fish for the last 20 min of the trial and the specificity of the preference was quantified as the interquartile range (IQR), that is, the temperature difference between the 75th and the 25th percentiles of the time spent at different temperatures during these last 20 min (see ref. ^43^ for more details).

### SMR

Standard metabolic rate trials started on the day following the thermal preference trials for all eight fish in a group that had been removed from their holding tanks and starved 2 days prior (four of these fish were tested for thermal preference the day before). SMR was measured in two four-chamber intermittent-flow respirometry systems (Loligo Systems), allowing the testing of eight fish simultaneously. The two systems contained eight cylindrical glass chambers (~0.018 l) with contactless sensor spots (PyroScience) on the inner wall. Eight fibre-optic oxygen sensors (PyroScience) were pointed towards the sensor spots and connected to two four-channel oxygen meters (Firesting-O2 and FireSting-Pro, PyroScience) recording to oxygen-logging software (Pyro Oxygen Logger and Pyro Workbench, PyroScience). A temperature probe connected to the oxygen meters was placed in both systems. Before each trial the setup was filled with UV-treated air-saturated water at 28 °C and a plastic syringe was used to ensure that there was no air in the pumps or tubing. The temperature in both systems was controlled by circulating the water to a common water bath where the water was aerated. Recirculation pumps connected to each chamber ensured mixing in the chambers and a flush pump replenished the chambers with the water from the surrounding system. The oxygen sensors were calibrated to air-saturated water daily when the system was running at a stable temperature.

Twenty-four groups of eight fish were tested for SMR. At the start of each trial, eight fish were placed in individual chambers, and the chambers were closed. The respiration (MO_2_) was recorded in intervals during which the flush pumps were turned off and the oxygen level in the closed recirculation circuit dropped due to the fish respiration. The fish were left overnight (16 h) at 28 °C with flushing at regular intervals (4 min recirculation and 5 min flushing). The following morning one fish was removed from the setup to obtain a measurement of background respiration (oxygen consumption not caused by the fish) from that chamber. To measure the response of SMR to a temperature increase, the temperature was brought to 34 °C over 1 h (adjusted 1 °C every 10 min), starting at 9:00. Fish were kept at 34 °C and the respiration was recorded for three more hours. All fish were then removed from the setup, anaesthetized, identified, weighed and measured, before being transferred to a tank with the rest of their group. A final background measurement was taken in all chambers at 34 °C.

Between each trial, the glass chambers with sensor spots were removed from the setup and rinsed in ethanol (70%). The rest of the setup was cleaned with chlorine (1:100) while the system was running for 20 min before it was emptied, thoroughly rinsed with water and refilled. The respR package was used to correct for background respiration and convert all MO_2_ data^44^. To correct the 28 °C data for background respiration (that is, any respiration not associated with the fish), we assumed zero background respiration at the start and a linear increase to the average background measured at the end of all 28 °C trials (with no fish in the chamber). Background respiration was subtracted from each measurement using this back calculation. The empty chamber at 34 °C was used to estimate the average slope of increasing background respiration, and this was combined with the individual final background measurements to subtract the background rate from the recorded data. Individual SMR was estimated using the quantile with *P* = 0.2 (that is, 20% of the data below this point) from the values of MO_2_ measurements following the recommendations from Chabot (2016) and the approach was examined to match the bands of low values in MO_2_ plots^45^.

Individual weight was taken after each trial. The mass statistically significantly affected the metabolic rate, and for direct comparison among individuals, we calculated the metabolic rate (mg O_2 _h^−1^) adjusted to the mean weight (105 mg) using the linear regression log_10_(SMR) = *b*_0_ + *b*_1_ × log_10_(weight), where *b*_0_ and *b*_1_ represent the intercept and slope of the scaling relationship. The mass-adjusted metabolic rate of each individual was then calculated as ${\rm{SMR}}_{\rm{adjusted}} = 10^{({\rm{b}}_{0}+{\rm{b}}_{1}\times{\rm{log}}_{10}({\rm{mean}}\, {\rm{weight}})+e)}$, where *e* is the individual’s residual value. The *Q*_10_ for SMR, the factorial change in metabolic rate over 10 °C, was calculated as $Q_{10}=(R_2/R_1)^{(10\,^\circ{\rm{C}}/T_2-T_1)}$, where *R*_1_ and *R*_2_ are the mass-adjusted SMR at the two temperatures *T*_1_ and *T*_2_ (28 °C and 34 °C).

### Maximum swim speed

On the final day of phenotyping, four fish from each group (the same that were tested for thermal preference) were individually tested for maximum swimming speed in a Blazka-type swim respirometer (1.5 l, Loligo Systems). Fish were first tested at 28 °C and then at 34 °C later the same day. The water temperature was maintained constant by circulating the water to an external heating bath. An individual was transferred to the swim chamber at the respective trial temperature (28 °C or acute transfer to 34 °C) and left to swim for 10 min at the lowest speed for steady swimming (<0.01 m s^−1^). The speed was then increased and kept at 0.04 m s^−1^ followed by 0.06 m s^−1^ for 5 min each. To estimate the maximum swimming speed, the speed of the flow was slowly increased until the fish could no longer maintain its position in the flume. The flow was then reduced, and the fish got 30 s of rest at 0.04 m s^−1^ before another maximum swimming speed test was performed. The highest speed of the two attempts was used as the maximum swimming speed of the individual. The speed was converted to bl s^−1^ using the length of the fish measured the day before the swimming speed test.

### Group MMR

The eight fish in each group were also tested for MMR in a custom-built (0.4 l) respirometer (6 × 14-cm circular glass container, +365, IKEA). The respirometer contained a steel mesh creating a bottom compartment for a stir bar, and a plastic pipe (4-cm diameter) in the middle to create a swimming arena in the outer circumference. It was placed in an insulated tank on a magnetic stirrer and the water in the tank was circulating through a heating bath to maintain a steady temperature during the trials. A hole in the lid of the respirometer was fitted with a rubber stopper with a glass chimney extending above the water surface. An oxygen probe (OXROB10, PyroScience) was inserted in the chimney and the oxygen level was recorded by an oxygen meter (FireSting-O2, PyroScience) and logged to a computer with logging software (Pyro Oxygen Logger, PyroScience). The eight fish were divided into two groups and tested first at 28 °C and then at 34 °C later the same day. The fish were transferred to the respirometer with air-saturated water at the respective testing temperature. The speed of the water was controlled by regulating the magnetic stirrer and the trial started with 5 min at 0.03 m s^−1^. Next, the fish were exposed to two intermediate speeds (0.04 and 0.06 m s^−1^) for 5 min each. Following this, the speed was slowly increased to the highest speed that all fish could maintain. When one fish could not keep its position in the current, the flow speed was reduced slightly and kept at the maximum speed the group could maintain for 5 min to measure the maximum oxygen consumption of the group. The fish were moved back to their holding tanks after the trials and background respirometry measurements were taken. Background correction was done using the respR package in R^44^. The mass measurement from the day before the MMR trial was used to calculate the total mass of the fish tested in each trial and both metabolic rate and weight were divided by the number of fish in the trial to calculate the mass-adjusted MMR (mg O_2_ h^−1^) as described above for SMR. The *Q*_10_ for mass-adjusted MMR was calculated using the same equation used for SMR. Aerobic scope (the residual metabolic capacity above basal metabolic requirements) was calculated by subtracting the mean-adjusted SMR of the fish in the group tested together from the mass-adjusted MMR.

### Acute thermal tolerance (CT_min_ and CT_max_)

Cooling and warming tolerance were assessed as the acute critical thermal minimum and maximum (CT_min_ and CT_max_) 5 days after the phenotyping ended (Supplementary Fig. 4). The CT_max_ test followed the same protocol as the one used during the selection experiment^19^. Both acute critical limits were tested with a temperature change of 0.3 °C per minute in a tank (25 × 22 × 18 cm) consisting of a small compartment separated by a mesh wall from a larger compartment for the fish. A 300 W heater and a circulation pump (Universal 300, Eheim) were placed in the small compartment in the CT_max_ trials, and the setup was filled with 11 l of water. The CT_min_ was tested in the same setup and the protocol was designed to correspond to the CT_max_ test. In the CT_min_ test, the water pump in the small compartment was connected to a cooler (Titan 500, Aqua Medic) placed below the setup. During the trials, water was pumped through the cooler and returned to the small compartment through hoses. To ensure a steady cooling rate, the flow rate through the cooler was increased three times during the trials using regulating valves on the returning water to the setup. The volume of water in the tank was 10 l (1 l less than in the CT_max_ setup to ensure a steady rate of cooling) and an additional pump in the small compartment ensured the mixing of water to obtain homogenous temperature in the two compartments. In both CT_max_ and CT_min_ trials, fish were tested in groups from their respective holding tank (first measurement period: *n* = 5–8; second measurement period: *n* = 9–12). The temperature was ramped and continuously monitored, and the endpoint for both upper and lower acute thermal tolerance limit was defined for each individual when the fish lost equilibrium and experienced uncontrolled swimming for 3 s. At this point, the individual was netted out and its thermal limit was noted. All fish were tested for CT_min_ first and for CT_max_ 3 days later. After the CT_max_ trials, fish were euthanized (MS-222 overdose) and the final weight and length measurements were taken. Growth rates were calculated as proportional weight (*w*) increase per day (SGR) using the equation SGR = (ln(*w*_2_) − ln(*w*_1_))/days × 100 (ref. ^46^).

### HSP expression

Due to the expanded thermal tolerance scope with selection for increased warming tolerance, we performed an additional experiment to test the expression of HSP at baseline levels and after a heat shock mimicking the CT_max_ trials. This was tested by performing western blots during the autumn of 2023 on samples of fish (3 years old) from the same generation as the fish used for other phenotyping. Baseline HSP70 levels were measured for 12 fish from each replicated selection line by collecting fish from their holding tanks (28 °C) and immediately euthanizing the fish (MS-222 overdose). To test expression after heat shock, 12 fish from each replicated selection line were transferred to a CT_max_ setup (described above) at 28 °C. The temperature was ramped to 38 °C at 0.3 °C per minute and held at 38 °C for 10 min to mimic the total warming exposure duration of the CT_max_ measurements before the fish were euthanized. Both baseline and heat-shocked fish were weighed before brains were dissected out and the tissue was immediately transferred to 1.5-ml tubes containing 200 μl of ice-cold lysis buffer (RIPA, Thermo Scientific). All equipment (scalpel, forceps, scissors) was sterilized and washed with ethanol between each dissection.

The tissue was homogenized in the buffer using a plastic pestle, and then the protein was isolated by centrifuging (500*g*) for 5 min at 4 °C and collecting the supernatant (lysate) into a new set of tubes (1.5 ml). The total protein concentration of each lysate was determined by performing a Bicinchoninic acid assay^47^. The volume of lysate was adjusted to achieve a protein concentration of 1 μg μl^−1^ and a total volume of 100 μl by mixing with 4× Laemmli buffer (375 mM Tris-HCl, 9% SDS, 50% glycerol, 9% β-mercaptoethanol, 0.03% bromophenol blue; Thermo Scientific). Samples were boiled at 100 °C for 5 min.

For the western blots, we loaded 20 μl of each sample on 10% precast polyacrylamide gels (Bio-Rad). The gels were run at 150 V and 200 mA for 55 min in an electrophoresis cell (Mini-PROTEAN Tetra Vertical, Bio-Rad). The protein was transferred at 25 V and 1.3 A for 7 min (Trans-Blot Turbo Transfer System, Bio-Rad) to polyvinylidene fluoride membranes. After transfer, the membrane was washed with TBS-T (TBS: 25 mM Tris, 2.7 mM KCl, 137 mM NaCl, 0.1%; Tween: pH 7.4) followed by Ponceau S (Thermo Scientific) staining for 3 min and photographing to visually confirm equal lane loading (Syngene).

Each membrane was blocked with 15 ml TBS-T with 5% milk powder for 1 hour at room temperature, washed with TBS-T and incubated with a 15 ml 1:1,000 of primary HSP70 antibody (ab210559) for 4 hours at 4 °C, followed by 15 ml of the 1:2,000 secondary antibody (ab288151) for 1 hour at room temperature. The membranes were imaged by adding enhanced chemiluminescence substrate (SuperSignal West Pico, Thermo Scientific) and an exposure time of 10 s. The relative HSP70 expression was quantified as integrated density of the HSP70 bands in the image (sum of the intensity of the pixels; ImageJ, v.1.53) and equal lane loading was visually confirmed by the Ponceau S stained pictures. The HSP70 levels are presented as relative to the Control line at baseline.

### Statistical analyses

Data were processed and analysed using R v.4.1.3 (ref. ^48^). All traits were analysed with linear mixed-effect models. Separate models were fitted for the traits tested at two different temperatures (SMR, MMR and maximum swim speed). Selection treatment was a fixed effect while holding tanks nested within replicate lines were included as random factors. When the measurements were divided into two periods, the measurement period was included as a fixed effect to account for the 24-day difference of the tested animals. Scaling relationships between body mass and SMR or MMR were assessed with models fitted on log_10_ transformed data where the predictor variable (log_10_ transformed body mass) was mean-centred. To assess the arithmetic means and contrasts between treatments for SMR and MMR, separate models were fitted on the mass-adjusted SMR and MMR (see methods for mass adjustment above). Models analysing data on egg size from the F_6_ generation and HSP from the F_7_ generation did not include the holding tank as a random factor. The model for egg size included the group of eggs photographed together nested within the spawning box and replicate line as a random factor, and the model for HSP included the order of testing as fixed factor. All models were fitted with maximum likelihood using the lme4 package^49^. We performed model selection using the Akaike information criterion corrected for small sample size. The best models were refitted with restricted maximum likelihood to obtain parameter estimates. We assessed the statistical significance of the different factors by considering if the 95% CI of the contrasts between groups overlapped with zero. Reported values in the text are means of parameter estimates and contrast between treatments with their 95% CI if not specified otherwise. Means are reported with standard error (s.e.) on the figures.

### Reporting summary

Further information on research design is available in the Nature Portfolio Reporting Summary linked to this article.

## Online content

Any methods, additional references, Nature Portfolio reporting summaries, source data, extended data, supplementary information, acknowledgements, peer review information; details of author contributions and competing interests; and statements of data and code availability are available at 10.1038/s41558-025-02332-y.

## Supplementary information

Supplementary Information Supplementary Figs. 1–4 and Tables 1–16.

Reporting Summary

## Data Availability

The data collected and used in this study are available via figshare at 10.6084/m9.figshare.28435322 (ref. ^50^).

## Code availability

The R code for the analyses of this study is available via figshare at 10.6084/m9.figshare.28435322 (ref. ^50^).
