# Supplementary material for: Evolution of warming tolerance alters physiology and life history traits in zebrafish
Source: Nat Clim Chang. 2025 May 14;15(6):665–72. doi: 10.1038/s41558-025-02332-y (PMC12158752; doi:10.1038/s41558-025-02332-y)
Supplement: Supplementary file 1 — Supplementary Figs. 1–4 and Tables 1–16. [file 41558_2025_2332_MOESM1_ESM.pdf]

nature climate change

Supplementary information https://doi.org/10.1038/s41558-025-02332-y

Evolution of warming tolerance alters physiology and life history traits in zebrafish

In the format provided by the authors and unedited

Supplementary figures

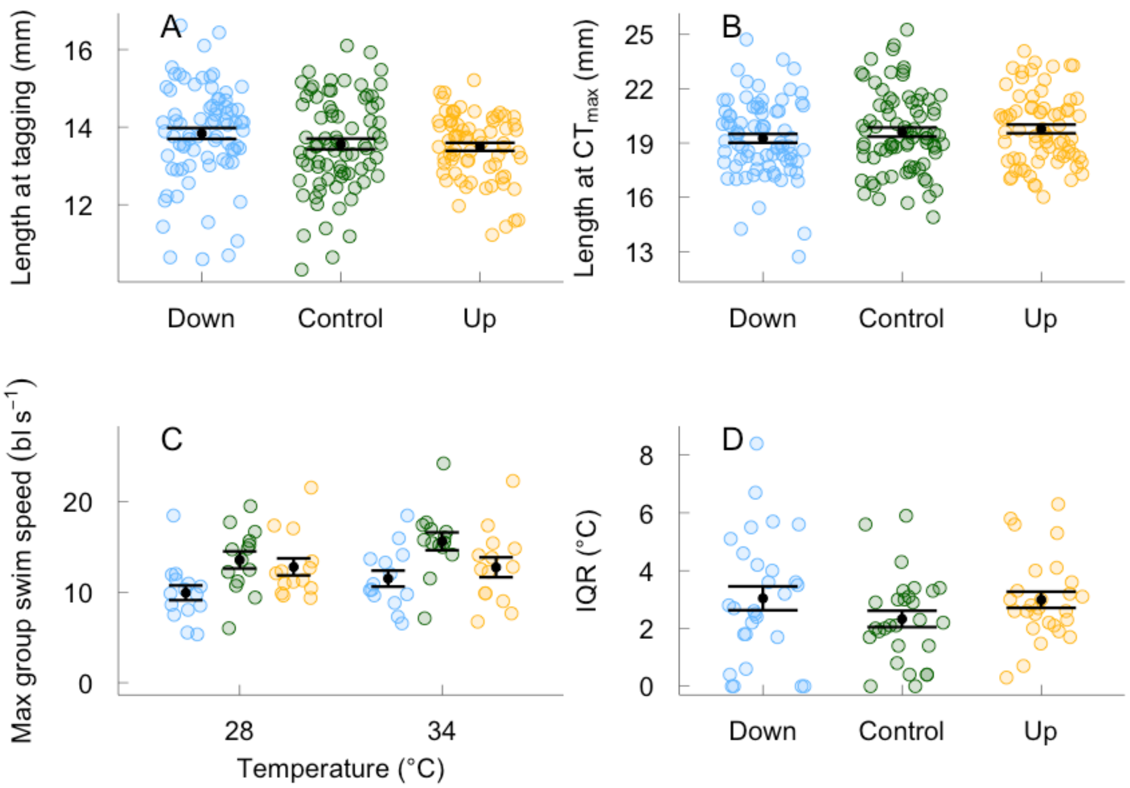

Figure S1 Effects of selection treatments on size, swim speed and thermal preference. A Standard length at tagging (*n* = individuals: Down = 78, Control = 78, Up = 70) and **B** at final sampling (*n* = individuals: Down = 78, Control = 78, Up = 70), **C** maximum swimming speed of groups at 28°C and 34°C (*n* = groups at 28°C: Down = 15, Control = 14 Up = 14; and at 34°C: Down = 14, Control = 14, Up = 14), and **D** the span of temperatures occupied in the thermal preference setup expressed as the interquartile range (IQR) of the distribution (*n* = individuals: Down = 28, Control = 28, Up = 27). Data are presented with mean (black points) ± standard error (error bars).

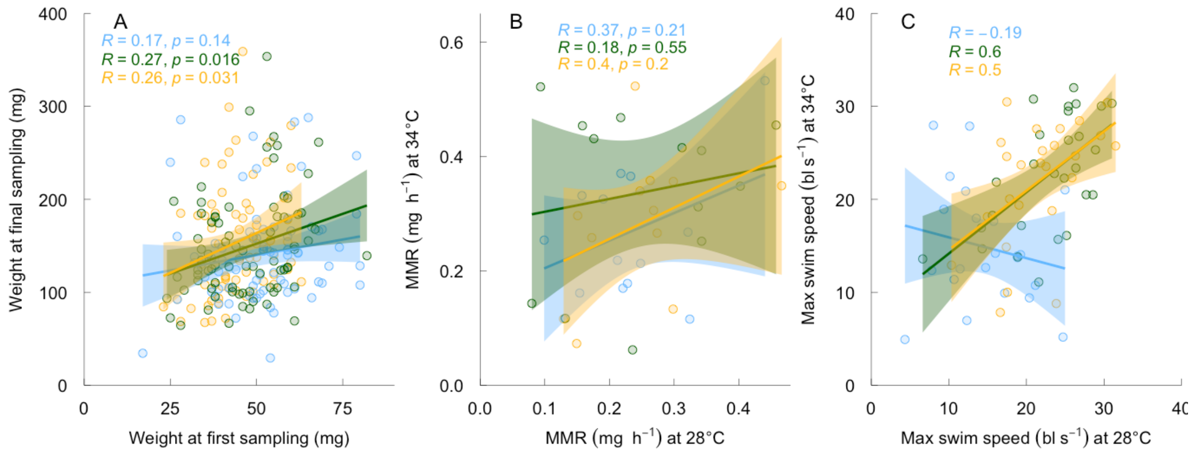

Figure S2 Relationships between traits in the different selection treatments. Relationship between **A** weight at first (44-47 dpf) and last (74-100 dpf) sampling (n = individuals: Down = 78, Control = 77, Up = 70), **B** MMR at 28°C and 34°C adjusted to a mean mass of 105 mg (n = groups: Down = 13, Control = 13, Up = 12), and **C** maximum swim speed at 28°C and 34°C in the three selection treatments (n = individuals: Down = 22, Control = 27, Up = 25). Regression lines represent the relationship between variables, with the shaded area indicating 95% confidence intervals.

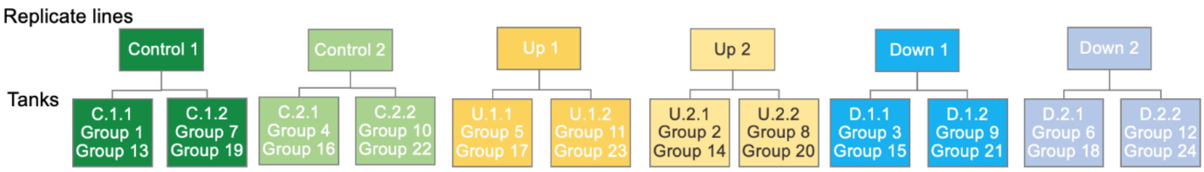

Figure S3 Design of the holding conditions for the phenotyped fish. The illustration represents the structure of the selection experiment with six duplicated selection lines, divided into two holding tanks with 20 fish in each. Two groups of eight fish were tested on separate days from each holding tank. Order of testing (1-24) was established to spread the selection treatments, replicate lines and holding tanks equally in the two phenotyping periods (see **Figure S4**).

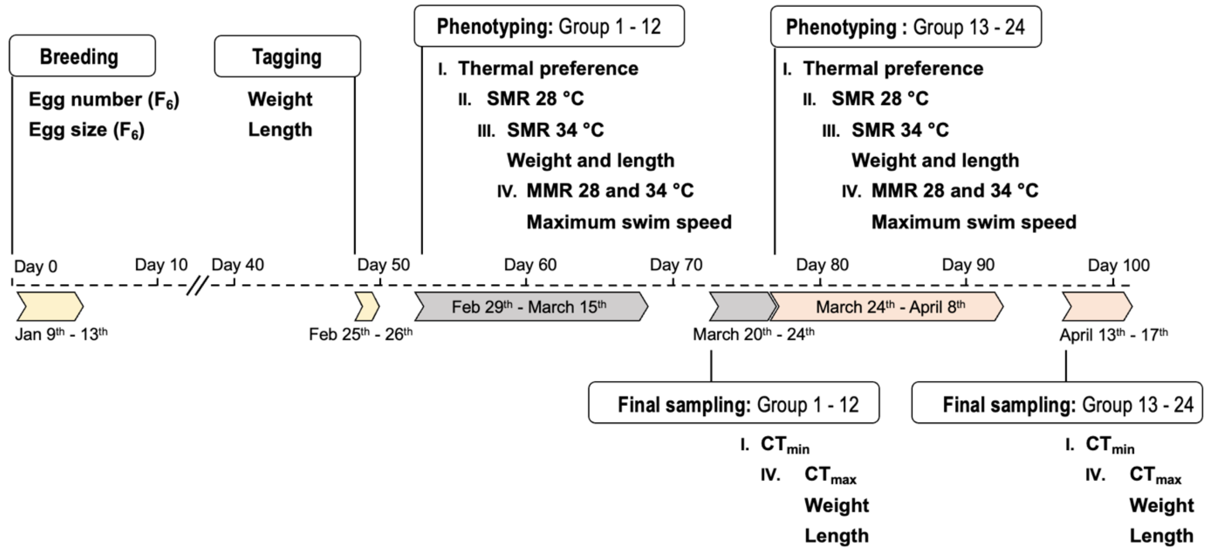

Figure S4 Timeline of the experiment. Indicates the dates for the different measurements. Roman numerals refer to the day of measurement within each phenotyping period.

Supplementary tables

Table S1 Results of the reproduction. Reproduction results at generation six for the different replicated lines in the three selection treatments. The column "Contributing tanks / Tanks" gives the number of tanks where reproduction was successful and the total number of attempts. The higher number of attempts in the Down-selected lines was due to the poor success of reproduction in these lines. The column "Fertilized eggs / Total eggs" gives the number of fertilized eggs and the total number of eggs in all contributing tanks and the final column gives the percentage fertilized eggs.

Line	Contributing tanks / Tanks	Fertilized eggs / Total eggs	% successfully fertilized
**Down 1**	6 / 12	239 / 501	48
**Down 2**	4 / 31	345 / 863	40
**Control 1**	5 / 7	1094 / 1524	72
**Control 2**	6 / 7	181 / 375	48
**Up 1**	6 / 7	1057 / 1513	70
**Up 2**	5 / 7	202 / 529	38

Table S2 Experimental schedule for testing the groups for different traits.

Date	Hotels	Thermal preference	SMR 28	SMR 34, weight, length	MMR, maximum swim speed	CT_{min}	CT_{max}, weight, length
29 Feb	1						
1 Mar	2	1					
2 Mar	3	2	1				
3 Mar	4	3	2	1			
4 Mar	5	4	3	2	1		
5 Mar	6	5	4	3	2		
6 Mar	7	6	5	4	3		
7 Mar	8	7	6	5	4		
8 Mar	9	8	7	6	5		
9 Mar	10	9	8	7	6		
10 Mar	11	10	9	8	7		
11 Mar	12	11	10	9	8		
12 Mar		12	11	10	9		
13 Mar			12	11	10		
14 Mar				12	11		
15 Mar					12		
20 Mar						1-6	
21 Mar						7-12	
22 Mar							
23 Mar							1-6
24 Mar	13						7-12
25 Mar	14	13					
26 Mar	15	14	13				
27 Mar	16	15	14	13			
28 Mar	17	16	15	14	13		
29 Mar	18	17	16	15	14		
30 Mar	19	18	17	16	15		
31 Mar	20	19	18	17	16		
1 Apr	21	20	19	18	17		
2 Apr	22	21	20	19	18		
3 Apr	23	22	21	20	19		
4 Apr	24	23	22	21	20		
5 Apr		24	23	22	21		
6 Apr			24	23	22		
7 Apr				24	23		
8 Apr					24		
13 Apr						13-18	
14 Apr						19-24	
15 Apr							
16 Apr							13-18
17 Apr							19-24

Table S3 Model selection for thermal tolerance models. The ΔAIC column represents the difference in AIC between each model and the top-ranked model and k is the number of parameters estimated. All models include *Tank* nested within *Replicate* as random effects. The best model is presented in bold.

	Model	k	AICc	ΔAIC
1	**CT_{max} ~ Treatment × Period**	**9**	**237.5**	**0**
2	CT_{max} ~ Treatment + Period	7	244.9	7.39
3	CT_{max} ~ Treatment	6	249.5	11.99
4	CT_{max} ~ Period	5	258.1	20.58
5	CT_{max} ~ 1	4	262.6	25.06
1	CT_{min} ~ Treatment × Period	9	278.1	1.86
**2**	**CT_{min} ~ Treatment + Period**	**7**	**276.2**	**0**
3	CT_{min} ~ Treatment	6	281.9	5.66
4	CT_{min} ~ Period	5	281.7	5.47
5	CT_{min} ~ 1	4	287.7	11.45
1	**Thermal scope ~ Treatment × Period**	**9**	**429.7**	**0**
2	Thermal scope ~ Treatment + Period	7	430.6	0.93
3	Thermal scope ~ Treatment	6	441.4	11.71
4	Thermal scope ~ Period	5	443.8	14.13
5	Thermal scope ~ 1	4	455.0	25.28

Table S4 Parameter estimates for thermal tolerance traits. The "diff" column reports the contrasts between the Control treatment and the Down or Up selected treatments, respectively. Parameters were estimated from the best models in **Table S3**. Contrasts significantly different from zero (zero not included in the 95% CI) are reported in bold.

		Control		Down				Up			
		Mean	SE	Mean	SE	diff	95% CI	Mean	SE	diff	95% CI
CT_{max} (°C)	Period 1	41.55	±0.08	40.88	±0.08	**-0.67**	**-0.91; -0.43**	41.77	±0.09	0.22	-0.02; 0.46
	Period 2	41.59	±0.07	41.28	±0.07	**-0.31**	**-0.53; -0.09**	41.75	±0.08	0.17	-0.03; 0.37
CT_{min} (°C)	Period 1	10.37	±0.10	10.5	±0.10	0.13	-0.14; 0.40	10.03	±0.10	**-0.35**	**-0.62; -0.08**
	Period 2	10.21	±0.10	10.33	±0.10	0.13	-0.14; 0.40	9.86	±0.10	**-0.35**	**-0.62; -0.08**
Thermal scope (°C)	Period 1	31.24	±0.14	30.4	±0.14	**-0.84**	**-1.21; -0.47**	31.68	±0.14	**0.43**	**0.04; 0.82**
	Period 2	31.34	±0.12	30.94	±0.12	**-0.4**	**-0.73; -0.07**	31.94	±0.12	**0.6**	**0.27; 0.93**

Table S5 Model selection for metabolic rate variables. The Δ*AIC* column represents the difference in AIC between each model and the top-ranked model, and the column *k* the number of model parameters. All models include *Tank* nested within *Replicate* as random effects. The best models are presented in bold. Models for SMR and MMR were fitted on the log_{10} transformed raw metabolic rate (mg h^{-1}) with mean-centred log_{10} transformed body weight as a fixed effect. Aerobic scope (AS) and thermal sensitivity (Q_{10}) were calculated from SMR and MMR adjusted to the mean body mass (105 mg). Parameter estimates for the different models are presented in **Tables S6**, **S7**, and **S8**. Note that for MMR at 34°C and AS at 34°C, two models were supported (ΔAICc <1) and they are both presented in bold. In both cases, the parameter estimates for the contrast between the Down-selected and the control lines were statistically significant (the 95% CI did not overlap zero) indicating that Down-selected lines had lower MMR and AS than the control lines at this temperature. Therefore, we present the model parameter estimates for the two best models of MMR at 34°C in **Table S6** and **S8**, and for AS at 34°C in **Table S7** and **S8**.

	Model	k	AICc	ΔAIC
1	log_{10}(SMR_28) ~ Treatment × log_{10}(Weight) + Period	10	-264.8	7.5
2	log_{10}(SMR_28) ~ Treatment + log_{10}(Weight) + Period	8	-267.0	5.4
3	log_{10}(SMR_28) ~ Treatment + log_{10}(Weight)	7	-268.7	3.6
4	log_{10}(SMR_28) ~ Treatment + Period	7	-145.6	126.7
5	log_{10}(SMR_28) ~ log_{10}(Weight) + Period	6	-270.7	1.6
6	log_{10}(SMR_28) ~ Treatment	6	-67.0	205.3
**7**	**log_{10}(SMR_28) ~ log_{10}(Weight)**	**5**	**-272.3**	**0.0**
8	log_{10}(SMR_28) ~ Period	5	-149.6	122.8
9	log_{10}(SMR_28) ~ 1	4	-71.1	201.2
1	log_{10}(SMR_34) ~ Treatment × log_{10}(Weight) + Period	10	-240.5	9.6
2	log_{10}(SMR_34) ~ Treatment + log_{10}(Weight) + Period	8	-244.3	5.8
3	log_{10}(SMR_34) ~ Treatment + log_{10}(Weight)	7	-246.3	3.8
4	log_{10}(SMR_34) ~ Treatment + Period	7	-146.0	104.1
5	log_{10}(SMR_34) ~ log_{10}(Weight) + Period	6	-248.2	1.9
6	log_{10}(SMR_34) ~ Treatment	6	-69.3	180.9
**7**	**log_{10}(SMR_34) ~ log_{10}(Weight)**	**5**	**-250.1**	**0.0**
8	log_{10}(SMR_34) ~ Period	5	-150.2	100.0
9	log_{10}(SMR_34) ~ 1	4	-72.7	177.4
1	log_{10}(MMR_28) ~ Treatment × log_{10}(Weight) + Period	10	2.1	13.0
2	log_{10}(MMR_28) ~ Treatment + log_{10}(Weight) + Period	8	-2.1	8.7
3	log_{10}(MMR_28) ~ Treatment + log_{10}(Weight)	7	-5.2	5.7
4	log_{10}(MMR_28) ~ Treatment + Period	7	-1.1	9.8
5	log_{10}(MMR_28) ~ log_{10}(Weight) + Period	6	-8.2	2.6
6	log_{10}(MMR_28) ~ Treatment	6	12.5	23.3
**7**	**log_{10}(MMR_28) ~ log_{10}(Weight)**	**5**	**-10.9**	**0.0**
8	log_{10}(MMR_28) ~ Period	5	-6.6	4.2
9	log_{10}(MMR_28) ~ 1	4	7.4	18.3
1	log_{10}(MMR_34) ~ Treatment × log_{10}(Weight) + Period	10	-4.6	9.5
2	log_{10}(MMR_34) ~ Treatment + log_{10}(Weight) + Period	8	-12.0	2.1
**3**	**log_{10}(MMR_34) ~ Treatment + log_{10}(Weight)**	**7**	**-14.1**	**0.0**
4	log_{10}(MMR_34) ~ Treatment + Period	7	-11.9	2.2
5	log_{10}(MMR_34) ~ log_{10}(Weight) + Period	6	-12.1	1.9

6	log_{10}(MMR_34) ~ Treatment	6	5.1	19.2
**7**	**log_{10}(MMR_34) ~ log_{10}(Weight)**	**5**	**-13.5**	**0.5**
8	log_{10}(MMR_34) ~ Period	5	-12.3	1.8
9	log_{10}(MMR_34) ~ 1	4	6.1	20.2
1	AS_28 ~ Treatment × Period	9	-75.2	10.1
2	AS_28 ~ Treatment + Period	7	-77.9	7.4
3	AS_28 ~ Treatment	6	-79.7	5.6
4	AS_28 ~ Period	5	-83.9	1.4
**5**	**AS_28 ~ 1**	**4**	**-85.3**	**0.0**
1	AS_34 ~ Treatment × Period	9	-67.2	10.1
2	AS_34 ~ Treatment + Period	7	-74.0	3.3
3	**AS_34 ~ Treatment**	**6**	**-76.7**	**0.6**
4	AS_34 ~ Period	5	-74.8	2.5
**5**	**AS_34 ~ 1**	**4**	**-77.3**	**0.0**
1	Q10_SMR ~ Treatment × Period	9	191.3	6.8
2	Q10_SMR ~ Treatment + Period	7	188.9	4.4
3	Q10_SMR ~ Treatment	6	187.7	3.2
4	Q10_SMR ~ Period	5	185.6	1.1
5	**Q10_SMR ~ 1**	**4**	**184.5**	**0.0**
1	Q10_MMR ~ Treatment × Period	9	114.8	11.2
2	Q10_MMR ~ Treatment + Period	7	111.4	7.8
3	Q10_MMR ~ Treatment	6	108.5	4.9
4	Q10_MMR ~ Period	5	105.8	2.2
5	**Q10_MMR ~ 1**	**4**	**103.6**	**0.0**

Table S6 Parameter estimates for metabolic rates. Standard metabolic rate (SMR) and maximum metabolic rate (MMR) measured at 28°C and 34°C. For each trait, the first row (log_{10}-log_{10}) presents the intercept and the slope (scaling exponent) from the best models (**Table S5**) fitted with log_{10} transformed metabolic rate (mg h^{-1}) and mean-centred log_{10} transformed body weight. The second row represents the arithmetic means (intercepts) from models on SMR and MMR (mg O_2 h^{-1}) adjusted to the mean weight (105 mg).

	Model	Intercept	SE	Scaling exponent	SE
	log_{10}-log_{10}	-1.30	±0.01	0.84	±0.04
SMR 28°C	mass adjusted (mg O_2 h^{-1})	0.05	±0.00		
	log_{10}-log_{10}	-1.08	±0.01	0.78	±0.04
SMR 34°C	mass adjusted (mg O_2 h^{-1})	0.09	±0.00		
	log_{10}-log_{10}	-0.81	±0.04	1.06	±0.203
MMR 28°C	mass adjusted (mg O_2 h^{-1})	0.17	±0.01		
	log_{10}-log_{10}	-0.72	±0.04	0.95	±0.17
MMR 34°C	mass adjusted (mg O_2 h^{-1})	0.20	±0.02		

Table S7 Parameter estimates for aerobic scope and thermal sensitivity of metabolic rates. Aerobic scope (AS) at 28°C and 34°C and thermal sensitivity (Q_{10}) of SMR and MMR. Both aerobic scope and Q_{10} were calculated from SMR and MMR adjusted to the mean mass (105 mg).

	Mean	SE
AS 28°C (mg h^{-1})	0.12	±0.01
AS 34°C (mg h^{-1})	0.11	±0.02
Q_{10} of SMR	2.24	±0.07
Q_{10} of MMR	1.61	±0.22

Table S8 Parameter estimates for maximum metabolic rate and aerobic scope at 34°C. The "diff" column reports the contrasts between the Control treatment and the Down- or Up-selected treatments, respectively. For maximum metabolic rate (MMR) at 34°C the first row (log_{10}-log_{10}) presents the intercept, effect of treatment, and the slope (scaling exponent) from the best model (**Table S5**) fitted with log_{10} transformed metabolic rate (mg h^{-1}) and mean-centred log_{10} transformed body weight. The second row represents the arithmetic means (intercepts) from models on MMR at 34°C (mg O_2 h^{-1}) adjusted to the mean weight (105 mg). The aerobic scope (AS) at 34°C was calculated from SMR and MMR adjusted to the mean mass (105 mg). Contrasts significantly different from zero (zero not included in the 95% CI) are reported in bold.

		Control		Down				Up				Scaling
		Mean	SE	Mean	SE	diff	95% CI	Mean	SE	diff	95% CI	exp.
MMR 34°C	log_{10}-log_{10}	-0.68	±0.05	-0.82	±0.05	**-0.14**	**-0.28; -0.004**	-0.65	±0.06	0.03	-0.12; 0.18	0.91
	mass adjusted (mg O_2 h^{-1})	0.23	±0.02	0.17	±0.02	**-0.06**	**-0.12; -0.01**	0.22	±0.02	-0.01	-0.07; 0.05	
AS 34°C	mass adjusted (mg O_2 h^{-1})	0.14	±0.02	0.08	±0.02	**-0.06**	**-0.12; -0.01**	0.12	±0.02	-0.03	-0.09; 0.03	

Table S9 Model selection for the effect of period and treatment. Models on weight at tagging and at CT_{max}, specific growth rate (SGR) and egg diameter. All models include *Image* nested within *Spawning box* nested within *Replicate* as random effects. The best models are presented in bold. The Δ*AIC* column represents the difference in AIC between each model and the top-ranked model, and the column *k* gives the number of parameters. Parameter estimates from the best models are presented in **Table S10** and **S11**.

	Model	k	AICc	ΔAIC
**1**	**WeightTagging~ Treatment**	**6**	**1761.1**	**0**
2	WeightTagging~ 1	4	1762.5	1.33
1	WeightCT$_{max}$~ Treatment × Period	9	2381.2	4.24
2	WeightCT$_{max}$~ Treatment + Period	7	2380.1	3.14
3	WeightCT$_{max}$~ Treatment	6	2474.8	97.82
**4**	**WeightCT$_{max}$~ Period**	**5**	**2376.9**	**0**
5	WeightCT$_{max}$~ 1	4	2471.5	94.57
1	SGR~ Treatment × Period	9	499.6	3.96
2	SGR ~ Treatment + Period	7	497.1	1.45
3	SGR ~ Treatment	6	513.4	17.71
**4**	**SGR ~ Period**	**5**	**495.7**	**0**
5	SGR ~ 1	4	512.0	16.35
**1**	**Diameter~ Treatment**	**7**	**-2291.2**	**0**
2	Diameter~ 1	5	-2290.0	1.22

Table S10 Parameter estimates for egg diameter and weight at tagging. The contrasts between the Control treatment and the Down- or Up-selected treatments are presented as percentage difference (%diff) and absolute difference (diff) with its 95% confidence interval. Note that *Treatment* was included in the best models for both traits although the contrasts between the Control- and Up- or Down-selected treatments were non-significant (95% CI overlap zero). This was due to the statistically significant difference between the Up-selected and the Down-selected treatment for weight at tagging (-7.64 mg, 95% CI: -14.91; -0.37) and egg diameter (0.06, 95% CI: 0.001; 0.12).

	Control		Down					Up				
	Mean	SE	Mean	SE	%diff	diff	95% CI	Mean	SE	%diff	diff	95% CI
Weight tagging (mg)	46.84	±2.58	51.93	±2.58	10.87	5.09	-2.06; 12.24	44.3	±2.62	-5.42	-2.54	-9.75; 4.67
Egg diameter (mm)	1.33	±0.02	1.28	±0.03	-3.76	-0.05	-0.10; 0.01	1.34	±0.03	0.75	0.01	-0.04; 0.07

Table S11 Parameter estimates for weight at final weighing and specific growth rate. The contrasts between periods 1 and 2 are presented as percentage difference (%diff) and absolute difference (diff) with its 95% confidence interval for final weight (Weight CT_{max}) and specific growth rate (SGR).

	Period 1		Period 2				
	Mean	SE	Mean	SE	%diff	diff	95% CI
Weight CT$_{max}$ (mg)	106.98	±6.94	173.87	±6.29	62.53	**66.89**	**54.95; 78.83**
SGR (% day^{-1})	2.95	±0.17	2.54	±0.17	-13.89	**-0.41**	**-0.59; -0.23**

Table S12 Model selection physiological traits. Models on maximal swim speed (body lengths s^{-1}), thermal preference (median occupied temperature), and the interquartile range of occupied temperatures (IQR), all including *Tank* nested within *Replicate* as random effects. Model selection for the effect of selection treatment and order of sampling (1-5) on heat shock protein (HSP70) level relative to the Control-line at baseline (Intensity_baseline), or after heat shock (Intensity_heatshock). Note that for HSP70 at baseline, several models were supported (ΔAICc <1). HSP models include *Replicate* as a random effect. Therefore, results from both the simplest model and the model including *Treatment* (both in bold) are presented in Tables S13 and Table S15. The best models are presented in bold. The Δ*AIC* column represents the difference in AIC between each model and the top-ranked model, and the column *k* gives the number of model parameters.

	Model	k	AICc	ΔAIC
1	MaxSpeed_28~ Treatment × Period	9	518.5	3.04
**2**	**MaxSpeed_28~ Treatment + Period**	**7**	**515.4**	**0**
3	MaxSpeed_28~ Treatment	6	517.1	1.66
4	MaxSpeed_28~ Period	5	522.6	7.18
5	MaxSpeed_28~ 1	4	525.7	10.25
1	MaxSpeed_34~ Treatment × Period	9	502.6	7.3
2	MaxSpeed_34~ Treatment + Period	7	497.7	2.37
**3**	**MaxSpeed_34~ Treatment**	**6**	**495.3**	**0**
4	MaxSpeed_34~ Period	5	503.1	7.8
5	MaxSpeed_34~ 1	4	500.8	5.5
1	Median ~ Treatment × Period	9	349.4	8.45
2	Median ~ Treatment + Period	7	344.6	3.64
3	Median ~ Treatment	6	346.8	5.85
**4**	**Median ~ Period**	**5**	**340.9**	**0**
5	Median ~ 1	4	343	2.08
1	IQR ~ Treatment × Period	9	343.2	7.41
2	IQR ~ Treatment + Period	7	339	3.21
3	IQR ~ Treatment	6	338.1	2.23
4	IQR ~ Period	5	336.9	1.02
**5**	**IQR ~ 1**	**4**	**335.8**	**0**
1	Intensity_baseline ~ Treatment × Order	8	9.9	7.23
2	Intensity_baseline ~ Treatment + Order	6	3.5	0.91
**3**	**Intensity_baseline ~ Treatment**	**5**	**3.0**	**0.41**
4	Intensity_baseline ~ Order	4	2.7	0.07
**5**	**Intensity_baseline ~ 1**	**3**	**2.6**	**0**
1	Intensity_heatshock ~ Treatment × Order	8	15.3	6.23
2	Intensity_ heatshock ~ Treatment + Order	6	14.0	4.91
3	Intensity_ heatshock ~ Treatment	5	13.9	4.82
**4**	**Intensity_ heatshock ~ Order**	**4**	**9.1**	**0**
5	Intensity_ heatshock ~ 1	3	9.4	0.26

Table S13 Parameter estimates for maximal swimming speed at 28°C and 34°C, and heat shock protein 70. The contrasts between the Control treatment and the Down or Up selected treatments are presented as percentage difference (%diff) and absolute difference (diff) with the 95% confidence interval. Contrasts significantly different from zero (zero not included in the 95% CI) are reported in bold.

			Control		Down					Up				
			Mean	SE	Mean	SE	%diff	diff	95% CI	Mean	SE	%diff	diff	95% CI
Max swim speed ($bl\ s^{-1}$)	28°C	Period 1	21.12	±1.60	12.5	±1.62	-40.83	**-8.62**	**-12.56; -4.68**	19.45	±1.68	-7.93	-1.68	-5.66; 2.30
		Period 2	24.00	±1.50	15.37	±1.51	-35.94	**-8.62**	**-12.56; -4.68**	22.32	±1.52	-6.98	-1.68	-5.66; 2.30
	34°C		22.51	±1.60	14.58	±1.72	-35.2	**-7.92**	**-12.53; -3.31**	21.84	±1.63	-2.97	-0.67	-5.16; 3.82
HSP70 baseline			1.00	±0.07	1.17	±0.06	17	0.17	-0.02; 0.35	1.19	±0.06	19	0.19	0.004; 0.37

Table S14 Parameter estimates for thermal preference. Thermal preference measured as the median occupied temperature in a thermal gradient. The contrasts between periods 1 and 2 are presented as absolute difference (diff) with the 95% confidence interval.

	Period 1		Period 2			
	Mean	SE	Mean	SE	diff	95% CI
Median (°C)	30.53	±0.52	29.24	±0.44	-1.29	-2.47; 3.07

Table S15 Parameter estimates for the range of occupied temperatures and heat shock protein 70. Range of temperatures quantified as the interquartile range of occupied temperatures (IQR) and brain HSP70 levels at normal holding temperatures (baseline).

	Mean	SE
IQR (°C)	2.76	±0.25
HSP70 baseline	1.12	±0.04

Table S16 Parameter estimates for brain heat shock protein 70. Heat shock protein 70 (HSP70) levels after heat shock and the effect of the order of sampling (1-5).

	Mean	SE	Order Effect	SE	95% CI
HSP70 heat shock	1.33	±0.12	0.05	±0.03	-0.01; 0.10
