# Supplementary material for: Evolution of warming tolerance alters physiology and life history traits in zebrafish
Source: Nat Clim Chang. 2025 May 14;15(6):665–72. doi: 10.1038/s41558-025-02332-y (PMC12158752; doi:10.1038/s41558-025-02332-y)
Supplement: Supplementary file 2 — Reporting Summary [file 41558_2025_2332_MOESM2_ESM.pdf]

nature portfolio

Corresponding author(s): Anna H. Andreassen

Last updated by author(s): Feb 18, 2025

Reporting Summary

Nature Portfolio wishes to improve the reproducibility of the work that we publish. This form provides structure for consistency and transparency in reporting. For further information on Nature Portfolio policies, see our Editorial Policies and the Editorial Policy Checklist.

Statistics

For all statistical analyses, confirm that the following items are present in the figure legend, table legend, main text, or Methods section.

n/a	Confirmed	
☐	☒	The exact sample size (*n*) for each experimental group/condition, given as a discrete number and unit of measurement
☐	☒	A statement on whether measurements were taken from distinct samples or whether the same sample was measured repeatedly
☐	☒	The statistical test(s) used AND whether they are one- or two-sided<br>*Only common tests should be described solely by name; describe more complex techniques in the Methods section.*
☐	☒	A description of all covariates tested
☐	☒	A description of any assumptions or corrections, such as tests of normality and adjustment for multiple comparisons
☐	☒	A full description of the statistical parameters including central tendency (e.g. means) or other basic estimates (e.g. regression coefficient) AND variation (e.g. standard deviation) or associated estimates of uncertainty (e.g. confidence intervals)
☐	☒	For null hypothesis testing, the test statistic (e.g. *F*, *t*, *r*) with confidence intervals, effect sizes, degrees of freedom and *P* value noted<br>*Give P values as exact values whenever suitable.*
☒	☐	For Bayesian analysis, information on the choice of priors and Markov chain Monte Carlo settings
☒	☐	For hierarchical and complex designs, identification of the appropriate level for tests and full reporting of outcomes
☐	☒	Estimates of effect sizes (e.g. Cohen's *d*, Pearson's *r*), indicating how they were calculated

Our web collection on statistics for biologists contains articles on many of the points above.

Software and code

Policy information about availability of computer code

Data collection	No previously unreported custom computer code or algorithm was used to collect data.
Data analysis	No previously unreported custom computer code or algorithm was used to analyse data.

For manuscripts utilizing custom algorithms or software that are central to the research but not yet described in published literature, software must be made available to editors and reviewers. We strongly encourage code deposition in a community repository (e.g. GitHub). See the Nature Portfolio guidelines for submitting code & software for further information.

Data

Policy information about availability of data

All manuscripts must include a data availability statement. This statement should provide the following information, where applicable:

- Accession codes, unique identifiers, or web links for publicly available datasets
- A description of any restrictions on data availability
- For clinical datasets or third party data, please ensure that the statement adheres to our policy

The data and R code underlying the analyses of this study are available at figshare (10.6084/m9.figshare.28435322).

Research involving human participants, their data, or biological material

Policy information about studies with human participants or human data. See also policy information about sex, gender (identity/presentation), and sexual orientation and race, ethnicity and racism.

Reporting on sex and gender	*Use the terms sex (biological attribute) and gender (shaped by social and cultural circumstances) carefully in order to avoid confusing both terms. Indicate if findings apply to only one sex or gender; describe whether sex and gender were considered in study design; whether sex and/or gender was determined based on self-reporting or assigned and methods used. Provide in the source data disaggregated sex and gender data, where this information has been collected, and if consent has been obtained for sharing of individual-level data; provide overall numbers in this Reporting Summary. Please state if this information has not been collected. Report sex- and gender-based analyses where performed, justify reasons for lack of sex- and gender-based analysis.*
Reporting on race, ethnicity, or other socially relevant groupings	*Please specify the socially constructed or socially relevant categorization variable(s) used in your manuscript and explain why they were used. Please note that such variables should not be used as proxies for other socially constructed/relevant variables (for example, race or ethnicity should not be used as a proxy for socioeconomic status). Provide clear definitions of the relevant terms used, how they were provided (by the participants/respondents, the researchers, or third parties), and the method(s) used to classify people into the different categories (e.g. self-report, census or administrative data, social media data, etc.) Please provide details about how you controlled for confounding variables in your analyses.*
Population characteristics	*Describe the covariate-relevant population characteristics of the human research participants (e.g. age, genotypic information, past and current diagnosis and treatment categories). If you filled out the behavioural & social sciences study design questions and have nothing to add here, write "See above."*
Recruitment	*Describe how participants were recruited. Outline any potential self-selection bias or other biases that may be present and how these are likely to impact results.*
Ethics oversight	*Identify the organization(s) that approved the study protocol.*

Note that full information on the approval of the study protocol must also be provided in the manuscript.

Field-specific reporting

Please select the one below that is the best fit for your research. If you are not sure, read the appropriate sections before making your selection.

☐ Life sciences ☐ Behavioural & social sciences ☒ Ecological, evolutionary & environmental sciences

For a reference copy of the document with all sections, see nature.com/documents/nr-reporting-summary-flat.pdf

Ecological, evolutionary & environmental sciences study design

All studies must disclose on these points even when the disclosure is negative.

Study description	In this study, we used lines of fish descending from wild-caught zebrafish that had been artificially selected for seven generations towards higher or lower acute upper thermal tolerance. Each of the selected lines, Down, Control and Up, were kept in two replicated lines resulting in six replicated lines. Egg size and number were measured on embryos, while the rest of the phenotyping was done on fish between 43 and 100 days post fertilisation except for the measurements of heat shock protein expression measured on three-year-old fish of the same lines in an additional experiment.
Research sample	Egg size was measured on 320 eggs from the parental generation: a subset of 10 fertilized eggs were measured from 32 spawning events.<br>Further phenotyping: A total of 240 fish juvenile fish were tagged including forty juvenile F7 fish from each of the six selected lines. Twenty fish from the same line were housed together in 12 holding tanks with unique coloured tag combinations.<br>Heat shock protein expression: Total number of fish 72 was used and HSP70 expression at baseline and after heat shock were both measured for 12 fish from each replicated selection line.
Sampling strategy	In this study, we measured a range of traits on the same individuals. These traits were expected to have different variability and effect sizes. The sampling strategy was therefore chosen to sufficiently cover the structure of the data: Sampling 16 fish out of 20 per Tank leaving four extra fish in case of mortality, two tanks per replicated line and two replicated lines from each of the three selection treatments.
Data collection	Data was recorded by researchers and master's students at the Norwegian University of Science and Technology. The phenotypic traits measured were recorded in different experimental setups. The experiments included measuring thermal preference in an annular arena, metabolic rates in intermittent flow-through respirometry and closed respirometry, maximum swim speed in a swim flume, acute warming and cooling tolerance in custom made setups, and heat shock protein expression measurements from tissue samples. Further details on all the experimental procedures are provided in the methods section of the manuscript.
Timing and spatial scale	The main experiment and phenotyping were conducted in 2020. The fish were reproduced from January 9th and due to the Covid-19

Timing and spatial scale	outbreak, the fish from each tank went through phenotyping over two experimental periods (February 29th - March 15th and March 24th - April 8th). The order of testing ensured that eight fish from each tank were phenotyped before the lockdown and the remaining eight fish were tested 24 days later. The final sampling of the main experiment was done from April 13th to 17th. The additional experiment where heat shock protein expression was recorded, was performed during the fall of 2023.
Data exclusions	Statistical outliers were tested for and excluded if falling outside three times the interquartile range (IQR) below the Q1 or three times IQR above the Q3.
Reproducibility	Due to a break induced by the Covid-19 lockdown, experiments were conducted over two experimental periods. The effects of this break were tested for in all relevant analyses and results were found to be similar across the periods.
Randomization	The study design was ordered so that the testing of groups of eight fish (two per tank) were spread out across selection treatments, replication lines and replication tanks.
Blinding	Warming and cooling tolerance limits were determined by an experimenter blinded from the temperature in the setup, while an additional experimenter recorded the exact temperature for each individual. Other measurements of trait values were determined by processing data recordings from the lab, such as thermal preference from video recording or metabolic rates from oxygen measurements. These were not blinded but processed with automated analyses.

Did the study involve field work? ☐ Yes ☒ No

Reporting for specific materials, systems and methods

We require information from authors about some types of materials, experimental systems and methods used in many studies. Here, indicate whether each material, system or method listed is relevant to your study. If you are not sure if a list item applies to your research, read the appropriate section before selecting a response.

Materials & experimental systems

n/a	Involved in the study
☐	☒ Antibodies
☒	☐ Eukaryotic cell lines
☒	☐ Palaeontology and archaeology
☐	☒ Animals and other organisms
☒	☐ Clinical data
☒	☐ Dual use research of concern
☒	☐ Plants

Methods

n/a	Involved in the study
☒	☐ ChIP-seq
☒	☐ Flow cytometry
☒	☐ MRI-based neuroimaging

Antibodies

Antibodies used	Primary HSP70 antibody, ab210559 (Supplier Abcam) and secondary (Anti-rabbit) antibody, ab288151 (Supplier Abcam).
Validation	The primary antibody is tested on zebrafish (WB)

Animals and other research organisms

Policy information about studies involving animals; ARRIVE guidelines recommended for reporting animal research, and Sex and Gender in Research

Laboratory animals	In this study, we used lines of fish descending from wild-caught zebrafish that had been artificially selected for seven generations towards higher or lower acute upper thermal tolerance. Egg size and number were measured on embryos, while the rest of the phenotyping was done on fish between 43 and 100 days post fertilisation except for the measurements of heat shock protein expression measured on three-year-old fish of the same lines in an additional experiment.
Wild animals	This study did not involve wild animals.
Reporting on sex	Information on sex was not collected.
Field-collected samples	This study did not include samples collected from the field.
Ethics oversight	The experiment was approved by the Norwegian Animal Research Authority (permit number: 8578) and the experimental procedures were conducted in line with the Norwegian Animal Welfare Act and the Regulation on the Use of Animals in Research.

Note that full information on the approval of the study protocol must also be provided in the manuscript.

Plants

Seed stocks	*Report on the source of all seed stocks or other plant material used. If applicable, state the seed stock centre and catalogue number. If plant specimens were collected from the field, describe the collection location, date and sampling procedures.*
Novel plant genotypes	*Describe the methods by which all novel plant genotypes were produced. This includes those generated by transgenic approaches, gene editing, chemical/radiation-based mutagenesis and hybridization. For transgenic lines, describe the transformation method, the number of independent lines analyzed and the generation upon which experiments were performed. For gene-edited lines, describe the editor used, the endogenous sequence targeted for editing, the targeting guide RNA sequence (if applicable) and how the editor was applied.*
Authentication	*Describe any authentication procedures for each seed stock used or novel genotype generated. Describe any experiments used to assess the effect of a mutation and, where applicable, how potential secondary effects (e.g. second site T-DNA insertions, mosiacism, off-target gene editing) were examined.*
